# Epidemiology and Characteristics of *Elizabethkingia* spp. Infections in Southeast Asia

**DOI:** 10.3390/microorganisms10050882

**Published:** 2022-04-22

**Authors:** Asdren Zajmi, Jeanette Teo, Chew Chieng Yeo

**Affiliations:** 1 Centre for Research in Infectious Diseases and Biotechnology (CeRIDB), Faculty of Medicine, Universiti Sultan Zainal Abidin, Kuala Terengganu 20400, Malaysia; asza22@yahoo.com; 2 Faculty of Health and Life Sciences, Management and Science University, Seksyen 13, Shah Alam 40100, Malaysia; 3 Department of Laboratory Medicine, National University Hospital, Singapore 119074, Singapore; jeanette_teo@nuhs.edu.sg

**Keywords:** *Elizabethkingia* spp., antibiotic resistance, multidrug resistance, meningitis, bacteremia, outbreak, Southeast Asia

## Abstract

*Elizabethkingia* spp. is a ubiquitous pathogenic bacterium that has been identified as the causal agent for a variety of conditions such as meningitis, pneumonia, necrotizing fasciitis, endophthalmitis, and sepsis and is emerging as a global threat including in Southeast Asia. *Elizabethkingia* infections tend to be associated with high mortality rates (18.2–41%) and are mostly observed in neonates and immunocompromised patients. Difficulties in precisely identifying *Elizabethkingia* at the species level by traditional methods have hampered our understanding of this genus in human infections. In Southeast Asian countries, hospital outbreaks have usually been ascribed to *E. meningoseptica*, whereas in Singapore, *E. anophelis* was reported as the main *Elizabethkingia* spp. associated with hospital settings. Misidentification of *Elizabethkingia* spp. could, however, underestimate the number of cases attributed to the bacterium, as precise identification requires tools such as MALDI-TOF MS, and particularly whole-genome sequencing, which are not available in most hospital laboratories. *Elizabethkingia* spp. has an unusual antibiotic resistance pattern for a Gram-negative bacterium with a limited number of horizontal gene transfers, which suggests an intrinsic origin for its multidrug resistance. Efforts to prevent and further understand *Elizabethkingia* spp. infections and limit its spread must rise to this new challenge.

## 1. Introduction

The Gram-negative bacteria of the genus *Elizabethkingia* have recently emerged as an important pathogen in hospital-acquired infections and are generally associated with high mortality [1]. Recent literature has reported several cases of severe infection in humans owing to this organism, with neonatal meningitis most commonly presented in children [2], accompanied by a range of other clinical manifestations such as septicemia and bacteremia [3,4], osteomyelitis [5], urinary tract infections [6,7], endogenous endophthalmitis [8], endocarditis [9], epididymo-orchitis [10], pulmonary abscess [11], necrotizing fasciitis [12,13], cystic fibrosis [14], hydrocephalus [15], and secondary infections with a high mortality rate, particularly in immunocompromised patients [16]. *Elizabethkingia meningoseptica* infections have also been associated with COVID-19 patients [17]. *Elizabethkingia* spp. infects not only immunocompromised patients but also immunocompetent ones [18,19,20].

Historically, the first report of human infection due to *Elizabethkingia* was that of 19 cases of meningitis in infants in the United States of America [21]. Even in its earliest description, the isolates were demonstrated to be multidrug-resistant. Not long after King’s (1959) report, an outbreak of meningitis infection with *E. meningoseptica* was reported among neonates in the Congo [22] with varying sensitivities to chloramphenicol, carbomycin, magnamycin, and erythromycin.

Worldwide infections caused by *E. meningoseptica* were reportedly high amongst immunocompetent neonates as well as hospitalized patients with existing underlying infections, and in a comprehensive review, Dzuiban et al. [2] showed that from 283 cases reported from 28 countries from 1944 to 2017, 76% of them were neonates aged 0–1 month. From the 283 cases that were reviewed, 209 of the patients were diagnosed with meningitis [2]. Infections by this pathogen have been reported in many parts of the world, including in Southeast Asian countries such as Malaysia [2], Singapore [23], Thailand [24], Indonesia [25], and Cambodia [26]. However, until now, there have been no published reports from other Southeast Asian countries such as the Philippines, Brunei, Myanmar, Laos, and Timor-Leste. Although for Vietnam, there have been no published reports of clinical *Elizabethkingia* spp. infections, the isolation of the pathogen from the environment [27,28] suggests the existence of infections that could have been misidentified and/or have not been published. In Malaysia, there were only 32 cases from four published reports [2], indicating the scarcity of data for *Elizabethkingia* spp. infections in most countries around the region. The aim of this review is to summarize our current understanding of the characteristics of *Elizabethkingia* spp., the current epidemiological developments, and clinical manifestations of *Elizabethkingia* spp. in Southeast Asia.

## 2. Identification

When first discovered in 1959, the suggested name for the bacterium was *Flavobacterium meningosepticum*, which was later recommended to be changed to *Chryseobacterium meningosepticum* (in 1994) [29]. In 2005, it was assigned to the genus *Elizabethkingia* (named after the first scientist to report its’ discovery, Elizabeth King) under the Flavobacteriaceae family based on 16S rRNA phylogenetic studies [30]. Recently, whole-genome sequence analysis along with optical mapping and MALDI-TOF mass spectrometry led to the revision of the genus *Elizabethkingia* into eight species, namely *E. meningoseptica*, *E. miricola*, *E. anophelis*, *E. bruuniana*, *E. ursingii*, *E. occulta* [31], *E. argenteiflava* sp. nov. [32], and the latest *E. umeracha* [33].

Since correct identification of *Elizabethkingia* is difficult using traditional microbiological methods and misidentification of *E. anophelis* with *E. meningoseptica* has been found to be common (Lau et al., 2016), it is therefore highly likely for this pathogen to be underreported. Correct identification of the organism is crucial for the diagnosis and management strategies, as *E. anophelis* is a nososcomial pathogen [34]. Hence, differentiation between *E. anophelis* and *E. meningoseptica* requires accurate microbial identification, but the phenotypic similarities between *E. anophelis* and *E. meningoseptica* present a challenge to accurate identification, particularly for clinically derived isolates; 16S rRNA gene analysis had identified a 98.6% similarity between *E. meningoseptica* and *E. anophelis*, which has often led to the misidentification of these bacteria [34].

The four automated bacterial identification systems that are commonly used in diagnostic laboratories are: (1) API/ID32 Phenotyping Kits (bioMérieux, Marcy l’Etoile, France); (2) Phoenix 100 ID/AST Automated Microbiology System (Becton Dickinson Co., Sparks, MD, USA); (3) VITEK 2 Automated Identification System [35]; and (4) MALDI-TOF MS System (bioMérieux, Marcy l’Etoile, France) [36]. At the time of writing this review, the four microbial identification systems that are listed above do not, however, contain all eight species of *Elizabethkingia* in their reference spectra database. Studies have also shown that misidentification of *Elizabethkingia* was rife using these automated identification systems, with *E. anophelis* commonly misidentified as *E. meningoseptica* [1,35,37]. When the accuracy of the API/ID32, Phoenix 100 ID/AST, Vitek 2, and Vitek MS *Elizabethkingia,* clinical isolate identifications were compared with 16S rRNA gene sequencing; it was reported that species identification concordance between these identification systems and 16S rRNA gene sequencing was low at only 24.5–26.5% [35]. Nevertheless, MALDI-TOF MS systems with amended databases (labeled as “research-use only” system) either in the Vitek MS Knowledge Base v3.2 and Bruker MALDI Biotyper Library (Bruker Daltonics GmbH, Bremen, Germany) are now able to reliably differentiate *E. meningoseptica* from *E. anophelis*, but not the remaining species of the genus *Elizabethkingia* [35]. In a recent report of 22 clinical and 6 environmental hospital isolates from Queensland, Australia, Burnard et al. (2020) showed that the VITEK MS Knowledge Base v3.2 had a 96.2% accuracy in identifying *Elizabethkingia*, with a solitary isolate of *E. bruuniana* being the only species that was misidentified. Whole-genome sequencing confirmed that the majority of the isolates were *E. anophelis* (*n* = 22), with the rest being *E. miricola* (*n* = 3), *E. meningoseptica* (*n* = 2), and *E. bruuniana* (*n* = 1) [38].

In the near future, the inclusion of novel *Elizabethkingia* species spectra into the databases should ensure highly accurate identification using MALDI-TOF MS systems, making it a reliable identification tool in lieu of whole-genome sequencing.

## 3. Antibiotic Resistance

*Elizabethkingia* are intrinsically resistant to most β-lactams, β-lactam/lactamase inhibitors, and carbapenems due to the presence of two unique class B metallo-β-lactamases (MBLs), namely *bla*_BlaB_ and *bla*_GOB_, along with a class A extended-spectrum β-lactamase (ESBL), *bla*_CME_ [39,40,41]. *Elizabethkingia* are the only known bacteria thus far with multiple chromosomally encoded MBLs [42]. Reports of subclasses of MBL genes such as *bla*_BlaB-1_ [40], *bla*_BlaB_, and *bla*_GOB_ in both *E. meningoseptica* and *E. anophelis* [41], as well as *bla*_BlaB-16_ and *bla*_GOB-19_ in *E. miricola* isolated from a black-spotted frog in China [43], make *Elizabethkingia* spp. a possible environmental reservoir for β-lactam resistance.

*Elizabethkingia* isolates are frequently resistant to aminoglycosides, macrolides, tetracycline, and vancomycin but show variable susceptibility to piperacillin, piperacillin-tazobactam, fluoroquinolones, minocycline, tigecycline, and trimethoprim-sulfamethoxazole [3,38,41,44,45,46]; cephalosporins, monobactams, and moderate susceptibilities to piperacillin [47,48,49], ceftazidime, colistin, and meropenem [50]; and levofloxacin [51]. There are currently no established MIC breakpoints for *Elizabethkingia*, and susceptibilities are largely reported based on *Enterobacteriaceae* breakpoints of the Clinical and Laboratory Standards Institute (CLSI) M100 guidelines and/or the European Committee on Antimicrobial Susceptibility Testing (EUCAST) pharmacokinetic–pharmacodynamic (PK–PD) “non-species” breakpoints [37,38]. It has been pointed out that susceptibilities, especially for vancomycin and piperacillin-tazobactam as determined by disk diffusion and E-test, are deemed unreliable and inaccurate for *Elizabethkingia*, and broth microdilution is instead recommended for susceptibility determination [45,52]. Although successful therapy has been attributed to rifampicin, there has been a report of bacterial resistance after three days of starting treatment [53]. A similar case was reported for an *E. meningoseptica* isolate in the Kuala Lumpur General Hospital, which developed resistance during treatment to cefepime, a cephalosporin antibiotic that is normally highly active against both Gram-positive and Gram-negative organisms [54].

Using disk diffusion, Lau, Chow [1] reported 21 *Elizabethkingia* isolates from Hong Kong as susceptible to vancomycin. However, studies using broth microdilution tests on isolates from Taiwan [45,55] and Australia [38] indicated that the isolates are likely non-susceptible based on the high MICs obtained (that ranged between 8 and 256 µg/mL). Similar ranges of vancomycin MICs were obtained by Han et al. [37], who investigated *Elizabethkingia* isolates from South Korea using the agar dilution method and concluded that all isolates were non-susceptible based on the interpretive criteria used for *Staphylococcus* spp. The vancomycin resistance gene, *vanW*, was reported in the majority of *Elizabethkingia* genomes, although the exact function of *vanW* is currently unknown [38,46]. However, mutations in *vanW* have been identified in microorganisms with VanB-type glycopeptide resistance [46,56]. In view of these facts and despite some anecdotal reports of success in using intravenous vancomycin alone to treat *Elizabethkingia* infections [57,58], it was recommended that even if intravenous vancomycin is the favored therapy for *Elizabethkingia* meningitis, ciprofloxacin, linezolid, or rifampicin should also be included until future clinical studies could be carried out to conclusively determine the clinical efficacy of these vancomycin-combination regiments for treatment [52].

One of the earliest reports of the whole-genome sequences of *Elizabethkingia* spp. strains from Southeast Asia was from Singapore, whereby sputum isolates obtained from three patients (NUHP1, NUHP2, and NUHP3) and four from the hospital’s sink (NUH1, NUH4, NUH6, and NUH11) at the National University Hospital, Singapore, were compared against five previously sequenced *E. anophelis* strains Ag1 (PRJNA80705) and R26 (PRJNA178189), *E. meningoseptica* ATCC 12535 (NITE) (PRJNA199489), *E. meningoseptica* ATCC 12535 (OSU) (PRJNA198814), and *E. meningoseptica* 502 (PRJNA176121). This led to the discovery of 16 antibiotic resistance genes from the core genomes and 19 antibiotic resistance genes from the accessory genomes of *Elizabethkingia* spp., and this included genes that confer resistance to aminoglycosides, β-lactams, fluoroquinolones, glycopeptides, macrolide-lincosamide-streptogramins, tetracyclines, trimethoprim, and rifampicin [40]. A later study on two African isolates, E27017 and E18064, that compared their genomes with the genomes of 18 strains belonging to the genus *Elizabethkingia* from many different regions, including Malaysian and Singaporean genomic sequences that were available at that time, identified that all *Elizabethkingia* genomes contained at least 17 antimicrobial resistance genes (Supplementary Table S1) [41].

A whole-genome sequencing study on three isolates of *E. meningoseptica* collected from an outbreak from three separate patients living in different counties in the Midwest regions of Michigan led to the identification of 22 resistance genes and 18 multidrug resistance efflux pump-encoded genes (Supplementary Table S1) in all samples [59]. While *Elizabethkingia* spp. genomes shared many antibiotic-resistance genes with each other, minor differences have been reported [3,60]. Hence, genomic investigations of *Elizabethkingia* spp. offers invaluable novel information on the species, but unfortunately, there have not been any reports of the whole-genome sequence of *Elizabethkingia* spp. isolates from Southeast Asia besides those from Singapore.

## 4. Virulence Factors

**Figure 1:**
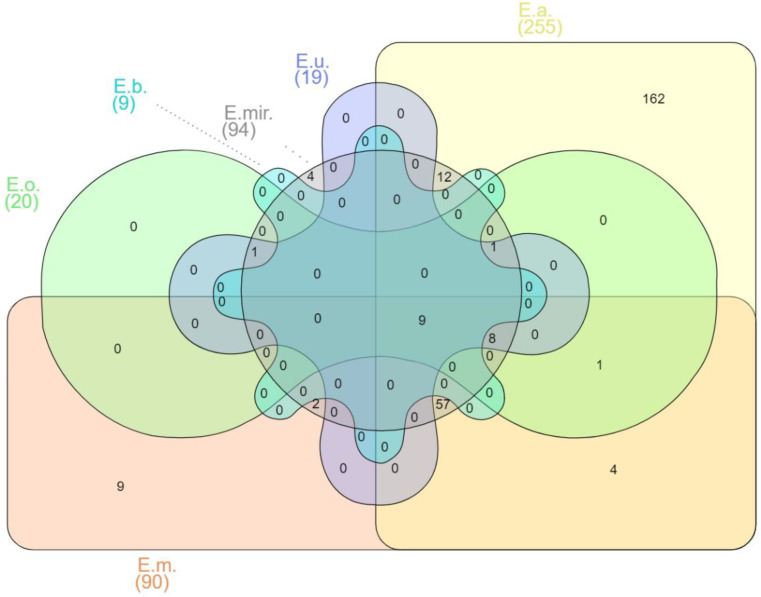
Venn diagram of shared virulence factor genes of *Elizabethkingia* spp. E.m.—*E. meningoseptica*; E.a.—*E. anophelis*; E.mir.*—E. miricola*; E.o.*—E. occulta*; E.u.—*E. ursingii*; E.b.—*E. bruuniana.* Edwards mode was used to process virulence factor gene outputs for Venn diagram visualization with InteractiVenn [74].

The mechanisms of pathogenesis of *Elizabethkingia* spp. are still being studied [59]. When the virulence factor database (VFDB, http://www.mgc.ac.cn/VFs/, accessed on 12 December 2021) was used to predict their presence from the genome sequences of various *Elizabethkingia* spp., this led to the prediction of a total of 270 putative virulence factor genes. More than fourteen virulence factor classes for *Elizabethkingia* spp. were identified (see Supplementary Table S2 for the complete list) with the following defined virulence-associated functions: adherence, antimicrobial activity, biofilm, cellular metabolism, effector delivery system, exoenzyme, exotoxin, immune modulation, invasion, motility, nutritional/metabolic factor, post-translational modification, regulation, stress survival, and others. Different species of *Elizabethkingia* shared the same virulence factors (Figure 1).

Among the 270 predicted genes for virulence factors, 162 have been reported as unique in *E. anophelis* (Supplementary Table S2). *E. meningoseptica* carried six unique genes involved in adherence that encode curli nucleator protein (*csgB*), curli assembly proteins (*curEm1, curEm2*, *curEm3*, *curEm4*), a curli production assembly protein (*csgG*), and two genes involved in immune modulation encoding a capsular polysaccharide synthesis enzyme (*cap8O*), a gene encoding Rab2-interacting conserved protein A (*ricA*) and a putative carbonic anhydrase-encoded gene (*mig-5*) (Supplementary Table S2). Four of the *E. miricola* unique virulence genes were predicted to be involved with urease accessory protein (*ureE*), urease alpha subunit (*ureA*), twitching motility protein (*pilG*), and sphingomyelinase-c (*smcL*) (Supplementary Table S2).

Identification of 6880 gene families in *E. anophelis* highlighted the genomic heterogeneity of *Elizabethkingia* species [41]. Genes homologous to heme iron acquisition, oxidative stress resistance proteins, and hemolysins were reported in earlier studies [34,61,62]. Extensive variations of capsular polysaccharide synthesis genes in *E. anopheles* were first reported by Breurec, Criscuolo [41], with variable *cps* clusters observed amongst the different lineages suggesting virulence heterogeneity among *Elizabethkingia* strains [41]. Identification of the capsule biosynthesis gene, *capD* [59], and the *adeG* gene for the AdeFGH efflux pump [20] in all *Elizabethkingia* species (Supplementary Table S2) leads to possible biofilm formation [44,63], which empowers the bacteria with the ability to persist on various surfaces [59,64]. Thirty clinical isolates from Malaysia, which comprised *E. anophelis*, *E. meningoseptica,* and *E. miricola,* were recently shown to produce biofilms on polystyrene microtiter plates [65].

Nine virulence factor genes were shared between six of the *Elizabethkingia* spp., including the *E. argenteiflava*-encoded *adeFGH* efflux pump, isocitrate lyase (*icl*), catalase/(hydro)peroxidase (*katA*), 60K heat shock protein (*htpB*), phospholipase C (*plc*), phosphopyruvate hydratase (*eno*), translation elongation factor (*tuf*), catalase/peroxidase HPI (*katG*), and aspartate 1-decarboxylase precursor (*panD*), which is involved with adherence, biofilm formation, cellular metabolism, exotoxin production, and stress survival (Supplementary Table S2). Isocitrate lyase (*icl*) plays an important role in the glyoxylate cycle [66], and its presence in *Elizabethkingia* spp. can predict its essential role in stationary-phase survival. An early report had shown that the presence of *icl* in *Mycobacterium tuberculosis* promoted the tenacity of infection by helping the pathogen to survive inside macrophages [67].

However, the specific role of bacterial enzymes in pathogenesis varies with infection. The presence of phospholipases C (*plc*) in all *Elizabethkingia* spp. [46] suggest its crucial role in downregulating host immunity [68]. In *L. monocytogenes*, *plc* aided bacterial escape toward the cytosol and cell-to-cell propagation, whereas, in *C. perfringens,* it helped bacteria induce endothelial damage and platelet aggregation, and in *P. aeruginosa*, it led to the triggering of signaling pathways that lead to inflammation [69]. 

The catalase-peroxidase genes, *katA* and *katG* (encoding hydroperoxidase I), are crucial against oxidative stress [70]. An earlier report showed that strains with *katA* were resistant to dodecyl sulfate, proteinase K, pepsin, trypsin, chymotrypsin, and the neutrophil protease cathepsin G, and they could survive for a long period once released from lysed cells [71]. Presence of *katA* [40,41,44,46,60,61,72,73] and *katG* [44,46,72,73] could also support *Elizabethkingia* species’ resistance to aminoglycosides.

## 5. Sources of Isolation and Transmission

The genera *Elizabethkingia* are aerobic, non-fermenting, non-motile, catalase-positive, oxidase-positive, indole-positive, and Gram-negative bacilli widely distributed in soil, mosquitoes, plants, fresh and marine fish [30,75], food products [76], hospital settings [77], stagnant water, inland wetlands, and rivers [33]. Due to their biofilm-forming ability [63], they have been isolated from sinks and taps where they colonize the most, leading to nosocomial and community infections [78] (Table 1).

Vector-borne transmission of the bacterial pathogen via mosquito bites has been suggested ever since the discovery of *E. anophelis* in the midgut of the *Anopheles gambiae* mosquito [79,80] and, more recently, in the salivary glands and saliva of *Aedes albopictus* [81]. The microbiome of *Anopheles* mosquitoes has evidently revealed the strong symbiotic nature of *E. meningoseptica* [82], which has been isolated from various independent sources, including *Anopheles stephensi*, the vector for the malarial parasite *Plasmodium vivax* [78,83,84], semi-field *Anopheles gambiae* females [82,84,85,86], field sampled mosquitoes in Cameroon [87,88], and laboratory-reared mosquitoes where *Anopheles* were the predominant species [87,89]. Another comparative study on bacterial microbiota isolated from the midgut of various *Anopheles* spp., which were obtained in the same region of Mae Sot District and Sop Moei District in Thailand, reported on the findings of *Elizabethkingia* spp. from *Anopheles minimus*, *Anopheles dirus, Anopheles maculatus, Anopheles sawadwongporni,* and *Anopheles dravidicus* mosquitoes [90]. However, sequences associated with the genus *Elizabethkingia* could not be definitively assigned to either *E. anophelis* or *E. meningoseptica* as the V3–V4 region of the 16S rRNA gene used for microbiome profiling could not differentiate between the two species [90]. Despite these multiple discoveries of *Elizabethkingia* spp. in the midgut and salivary glands of various mosquito species, there is currently a lack of strong direct evidence that supports *Elizabethkingia* infection, particularly *E. anophelis*, as a mosquito-borne disease [45], although this should not be ruled out with our current level of knowledge. A comparative genomics study of three cases of *E. anophelis* also provided evidence of vertical transmission from mother to her baby [62].

Zainuri et al. (2013) reported on the isolation of *E. meningoseptica* from American bullfrogs (*Lithobates catesbeianus* or *Rana catesbeiana*) suffering from red leg syndrome and cataract in Sabah, Malaysia [106]. Isolation of *E. meningoseptica* from bullfrogs was also described in an earlier study, in which the isolates obtained were found to be resistant to multiple antibiotics [107]. *E. miricola*, which had been implicated in acute infections in humans, caused a disease outbreak associated involving the internal organs of different anuran species, including northern leopard frogs (*Lithobates pipiens*), Chapa bug-eyed frogs (*Theloderma bicolor*), and Vietnamese warty toads (*Bombina microdeladigitora*) captured in Vietnam. The presence of β-lactamases and putative virulence genes in the *E. miricola isolates* were detected in silico [27].

*E. miricola* was also reportedly isolated from Tra catfish (*Pangasius hypophthalmus*) fillets in the industrial processing lines in Vietnam [109]. Tra catfish is a type of freshwater fish, which is one of the major fish species in the Mekong River, and its processed fillets are exported to more than 80 different countries worldwide [28]. Other scientists have also reported the isolation of *E. meningoseptica* from retail sausages in Kampar, Malaysia, although the identification was performed by traditional biochemical methods and identified as *Chryseobacterium meningosepticum* [76].

Furthermore, 454 pyrosequencing of the 16S rRNA gene from the bacterial community of the root of the gnetalean gymnosperm *Gnetum gnemon* and nearby bulk soils of a tropical forest arboretum at the Forest Research Institute of Malaysia (FRIM) at Kepong, near Kuala Lumpur, identified the mutualistic presence of *E. meningoseptica* and *E. miricola* [110]. *Elizabethkingia* spp. was surprisingly found in relative abundance (4.9%) on the leaves of *Gnetum gnemon* in comparison with rhizoplane (1.4%) [111]. These reports indicate the ubiquity of *Elizabethkingia* spp. in the environment and, thus, the difficulty in tracing an outbreak should one occur in the community and outside of hospital settings.

## 6. Malaysia Reports

Most reported cases of *Elizabethkingia* spp. infections in Malaysia occurred as isolated cases rather than outbreaks, and the most dominant strain is *E. meningoseptica* (Table 2). These early identifications of *E. meningoseptica* were made before laboratories could reliably distinguish between the different *Elizabethkingia* spp. Although there are currently no published reports on *E. anophelis* infections in Malaysia, whole-genome sequencing and assembly of a clinical isolate of *E. anophelis* B2D had been submitted to NCBI (Accession: PRJNA248328) by the University of Malaya but as *E. meningoseptica*. This led a group of scientists from the University of Malaya to revive thirty archived lyophilized isolates collected from their University Hospital that were initially identified as *Flavobacterium meningosepticum* [65]. Re-identification using 16S rRNA sequencing revealed that 24 were actually *E. anophelis,* whereas the remaining six were *E. miricola* [65]. None of the isolates were identified as *E. meningoseptica*, underlining the very high possibility of misidentification of these pathogens from earlier publications, particularly those that relied on traditional biochemical tests for their identification. Hence, the cases that had been previously reported as infections due to *E. meningoseptica* and reviewed below should be taken with caution.

The first case of *E. meningoseptica* (then reported as *Flavobacterium meningosepticum*) infection that was recorded in Malaysia involved three neonates with meningitis, where the infection was rapidly controlled by the use of rifamycin [97]. Another study reported six epidemiologically distinct isolates of *E. meningoseptica* (then reported as *Flavobacterium meningosepticum*) collected over a two-year period from neonates with meningitis in Kuala Lumpur [98]. From 1972 to 1981, the University of Malaya Medical Centre (UMMC) reported seven confirmed cases of *Flavobacterium meningosepticum* isolates in infants from the cerebrospinal fluid, three from the blood, and one from the peritoneal fluid [91]. Out of the seven infants, two of them died before receiving intraventricular chemotherapy, and the rest were treated with rifamycin SV, erythromycin, and novobiocin. Two of the surviving patients developed post-infection hydrocephalus, mental retardation, and spasticity (Thong et al., 1981). According to a prospective study carried out over a 12-month period (July 1986 and June 1987) among neonates positive for septicemia at the Special Care Nursery in Hospital Kuala Lumpur (HKL), 6 out of 155 were reported to be positive with *E. meningoseptica* infection and were treated with rifampicin, erythromycin, and novobiocin [112].

Another case study of resistant *E. meningoseptica* was confirmed at HKL and was successfully treated with the fourth-generation cephalosporin, cefepime [54]. Ali and Reddy (2007) reported an unusual finding of *E. meningoseptica* isolate in a bilateral simultaneous hypopyon corneal ulcer in a contact lens wearer caused by polymicrobial infection [101]. In addition, a study conducted at the adult ICU in Hospital Universiti Sains Malaysia (HUSM) involving 1869 organisms isolated in the period between 2005 and 2007 reported that 1% of the isolates comprised of *E. meningoseptica* [113]. In another study conducted at HKL, it was reported that out of five positive samples for microorganisms on CSF culture and sensitivity, one sample was positive with *E. meningoseptica* [99]. Septicemia due to *E. meningoseptica* in Malaysia was reported in a hemorrhagic stroke patient who developed septic shock during prolonged neuro-intensive care management at HUSM [100].

Findings from a study conducted at a 562-bed tertiary hospital in Selangor, Malaysia [102], showed that 4 out of 358 samples collected and analyzed were *E. meningoseptica*. Most of the isolates were from the surgical wards. Another study [15] reported on the isolation of *E. meningoseptica* from the blood and cerebrospinal fluid (CSF) of a premature infant of a dichorionic diamniotic (DCDA) twin with neonatal meningitis. The infant required non-invasive continuous pressure ventilation and, after 10 days, was noted to be febrile, less active, and developed seizures. The patient was successfully treated with intravenous vancomycin and ciprofloxacin for 6 weeks, and oral rifampicin was given for a total of 8 days according to the susceptibility testing of the organism. However, the patient’s recovery was complicated with hydrocephalus.

A prospective cohort study was conducted on gastrointestinal tuberculosis-suspected patients at the Queen Elizabeth Adult Hospital in Kota Kinabalu, Sabah, Malaysia [114]. Interestingly, blood cultures revealed the presence of *E. meningoseptica* in one of the cases, which was classified as a “non-tuberculosis case” using standard case definitions.

## 7. Singapore Reports

Although *E. anophelis* had been reported as the dominant species of *Elizabethkingia* in Singapore [92], it is not short of the presence of *E. meningoseptica*. Between April and June 2011, the National University Hospital (NUH) Singapore isolated three imipinem-resistant *E. meningoseptica* from rectal swabs of patients [105]. Another study by NUH reported an increasing prevalence of *E. meningoseptica* in ICUs after an environmental sampling control. About 44% (35/79) of the collected tap water samples were positive with *E. meningoseptica* [104].

During a three-week period in 2012, an investigation of an outbreak by the hospital infection control team at NUH showed that three cardiothoracic ICU patients and two surgical ICU patients were initially diagnosed with *E. meningoseptica* infection as identified by MALDI-TOF MS [115]. All patients were treated with intravenous piperacillin and tazobactam, cotrimoxazole, or levofloxacin, either alone or in combination; however, three of the patients succumbed to their infections due to septicemia. Three subsequent samples collected from the cardiothoracic ICU patients (NUHP1, NUHP2, and NUHP3) and four samples collected from the sink area (NUH1, NUH4, NUH6, and NUH11) were sent for whole-genome sequencing. Findings showed that the isolates obtained were more closely related to *E. anophelis* strains that were isolated from the midgut of the *Anopheles gambiae* malaria mosquito vector than to *E. meningoseptica* [40,115], thus making this the first case of *E. anophelis* outbreak in an ICU in Singapore.

Another case involved a 75-year-old patient with numerous comorbidities diagnosed with *E. meningoseptica* in all her four blood cultures after spending 2 months in the hospital for treatment of other conditions [8]. Three days after the diagnosis and starting treatment with intravenous cotrimoxazole, levofloxacin, and minocycline, she started developing redness and pain in her left eye with blurred vision. An intravitreal tap for vitreous culture was taken, and again, her results showed positive with *E. meningoseptica*. The patient was injected with intravitreal vancomycin and amikacin and had started on hourly fortified gentamicin and cefazolin eyedrops. The patient’s vitreous culture results showed that *E. meningoseptica* was susceptible to several antibiotics, including ciprofloxacin, levofloxacin, cotrimoxazole, and minocycline, but nevertheless resistant to ceftazidime, gentamicin, and amikacin. Thus, the intravitreal administration was switched to ciprofloxacin (100 µg/0.05 mL) and repeated five times. The patient’s anterior chamber fibrin clot progressively resolved and the inflammatory material in the vitreous cavity became organized, whereas vision was not recovered [8]. Another study also reported infections with carbapenem-resistant *E. meningoseptica* after 30 days of hospitalization at Tan Tock Seng Hospital (TTSH), Singapore [116].

Three patients aged 2.8 months, 4.9 months, and 4.8 years were diagnosed with *E. meningoseptica* using MALDI-TOF MS (VITEK MS) within 13 days in the ICU at Kandang Kerbau Women’s and Children’s Hospital (KKH), Singapore in 2017 [93]. Further investigation was conducted, and 27 environmental samples were collected from the three patients’ rooms or cubicles. Of 27 samples collected from tap water outlets and sinks, 10 samples showed positive with *E. anophelis,* and 1 sample was positive with *E. meningoseptica*. *E. meningoseptica* was isolated from a water tap not associated with any of the cases, and *E. anophelis* was isolated from an aerator [93].

In another retrospective study from 2009 to May 2017 conducted by Chew et al. (2018), seventy-nine blood isolates analyzed with Bruker MALDI Biotyper (bioMérieux) resulted in the identification of either *E. meningoseptica* (96.2%) or *E. miricola* (3.8%). Further 16S rRNA gene sequencing using universal primers was performed, and 77 samples showed closer nucleotide identity to *E. anophelis*. One sample each had a closer nucleotide identity to *E. anophelis* subsp. *Endophytica* and *E. meningoseptica* [92]. Due to the high resemblance between *Elizabethkingia* species, many isolates were initially misidentified as *E. meningoseptica* (Yung et al., 2018). Among the 77 isolates collected from hospital waste matters, the *bla*_SHV_-producing *E. meningoseptica* strain was identified among the most predominant taxa (1.4%), showing resistance to extended-spectrum cephalosporins and carbapenems [103].

An eight-year retrospective descriptive study (2010–2017) conducted in a tertiary pediatric hospital in Singapore reported 13 cases of patients with *E. meningoseptica* infection from the blood and 4 from CSF samples [23]. A 15.4% (2/13) mortality was reported among patients with *E. meningoseptica* bacteremia. However, a high (75%) number of morbidities consist of patients presented with meningitis. Most patients had developed post-infectious conditions such as hydrocephalus, quadriplegic cerebral palsy with severe disability, and reduced academic performance, whereas another had moderate global developmental delay.

A hemodialysis patient was confirmed positive for *E. meningoseptica* infection in a retrospective study conducted between January 2011 and June 2012 at the Singapore General Hospital (SGH). The patient was among 118 adult hemodialysis patients confirmed with vascular-access-associated bloodstream infection (VAABSI) [94].

Another case involved a preterm infant born with marked generalized abdominal distension and respiratory distress in the third trimester (33rd week), who was transferred to the neonatal intensive care unit for further care, and three weeks later, the patient was diagnosed with sepsis and subsequently, cloxacillin and amikacin were initiated. Blood samples were collected, and the patient was confirmed to be positive for *E. meningoseptica* infection. Antibiotic therapy was changed to rifampicin and piperacillin/tazobactam for a period of 4 weeks until full recovery [95].

Combined antibiotic therapy is reported to be the choice of treatment for *E. meningoseptica* patients in Singapore. The most commonly used combination is piperacillin/tazobactam with trimethoprim/sulfamethoxazole, followed by piperacillin/tazobactam with fluroquinolone [23,95]. Other antimicrobial agents used include minocycline [8,23], clindamycin, rifampicin, ciprofloxacin, cotrimoxazole, and levofloxacin [8].

Singapore and Wisconsin outbreak isolates have type I *cps* cluster [44]. Additionally, these outbreak strains carry a disrupted DNA repair *mutY* gene caused by the insertion of an integrative and conjugative element (ICEEa1). Genetic and morphological changes could have substantially contributed to the evolutionary dynamics of the outbreak strains that could have increased their concomitant adaptability eventuating in a hypermutator phenotype [44]. These “outbreak” strain features have not been studied in other Southeast Asian strains due to the lack of WGS investigation. It would not be surprising to find new Southeast Asian or geo-specific lineages, as was recently revealed by a distinct Taiwan strain [117].

## 8. Thailand Reports

In comparison with Malaysia and Singapore, there are not as many reports on *Elizabethkingia* infections from Thailand. Nevertheless, a retrospective study conducted by researchers from the University of Bangkok and Chulalongkorn University among eight hospitalized patients at King Chulalongkorn Memorial Hospital (KCMH) led to the first reported cases of *E. meningoseptica* infection in Thailand [24]. All isolates had shown resistance to cephalosporins, carbapenems, aminoglycosides, vancomycin, and colistin. Patients were treated with combined therapy of ciprofloxacin and cotrimoxazole, followed by levofloxacin, rifampicin, vancomycin, and imipenem. Despite the treatment, the mortality rate was high at 50% [24]. A subsequent report of MALDI-TOF analysis of 54 clinical isolates obtained from Siriraj Hospital, Mahidol University, showed that three of them were *E. meningoseptica* [36].

## 9. Indonesia Reports

Reports of *Elizabethkingia* spp. infections in Indonesia only appeared in the past couple of years. In Malang, Indonesia, a three-month-old infant presented to the Emergency Department of a tertiary hospital with a history of a 15-day fever associated with lethargy [13]. The patient was diagnosed with necrotizing fasciitis with cerebral salt wasting and disseminated intravascular coagulation. The patient had undergone fasciotomy and distal phalanges amputation. A postoperative blood sample revealed the presence of *E. meningoseptica* using the VITEK 2 system (bioMérieux). The isolate was further tested with various antimicrobials and showed susceptibility to cefepime, tigecycline, trimethoprim-sulfamethoxazole, intermediate susceptibility to combined antibiotics piperacillin-tazobactam, and resistance to ampicillin, ampicillin-sulbactam, cefazolin, ceftazidime, aztreonam, meropenem, amikacin, and gentamicin. The patient was put on three weeks cefepime (50 mg/kg per body weight/day) followed by mechanical ventilation, fluid and electrolyte therapy, intravenous hypertonic saline infusion, intravenous inotropic therapy, thrombocyte concentrate and fresh frozen plasma transfusion, and oral fluid cortisone therapy. After eight weeks of treatment, the patient showed clinical improvement.

In Jakarta, a group of researchers from the Eijkman Institute reported the presence of *E. meningoseptica* in one non-dengue febrile patient [118]. A retrospective cohort study conducted at the Pediatric Intensive Care Unit at Sanglah Hospital in Denpasar, Bali, Indonesia, from January 2015 to April 2017 led to the isolation of *E. meningoseptica* from one of their patients’ blood samples diagnosed with septicemia [25]. In a cross-sectional study conducted between April and August of 2015 in the neonatology ward of Haji Adam Malik Hospital in Medan, North Sumatra, 3 out of 43 neonates were found positive for *E. meningoseptica* from their blood samples, although the method of identification was not stated [96].

## 10. Cambodia Reports

Reports of *Elizabethkingia* spp. infections in Cambodia are rare. Reed et al. (2020) reported a case in which a 7-day-old female patient with presumed late-onset neonatal sepsis was transferred to the pediatric ICU at Angkor Hospital for Children, Siem Reap, Cambodia [26]. The patient had experienced symptoms of meningitis, including fever and seizures. Intravenous meropenem (40 mg/kg three times a day) was initiated, and after three days of hospitalization, blood culture isolated was identified as *E. anophelis* through MALDI-TOF MS using the bioMérieux VITEK MS. Later, the patient’s antibiotic therapy was replaced with intravenous ciprofloxacin (10 mg/kg, 2 per day) and vancomycin (15 mg/kg, 1 per day), and the patient was discharged after 28 days. A month later, the patient was hospitalized, and clinical features showed raised intracranial pressure, including neurologic deficits. The patient was later diagnosed with hydrocephalus. This had led the researchers to the retrieval of the seven isolates stored at −80 °C from January 2012 to October 2018 that had been previously identified as *C. meningosepticum*, *C. miricola*, or *Elizabethkingia* spp. Isolates were subcultured and analyzed using VITEK MS MALDI-TOF mass spectrometry; six of them were re-identified as *E. anophelis* and one isolate as *E. meningoseptica*. It is worth noting that four out of seven patients had died due to ventilator-associated pneumonia (VAP) and sepsis [26].

## 11. Conclusions

Our review of case reports involving *Elizabethkingia* spp. infections in Southeast Asia showed the dearth of knowledge in the majority of countries within the region. Of the eleven Southeast Asian nations, only Singapore had the most data and published reports, while we were unable to find any clinical case reports from the Philippines, Brunei, Vietnam, Laos, Myanmar, and Timor-Leste. Genomic data are also available mainly from Singapore, with currently only a single isolate of *E. anophelis* from Malaysia in GenBank (accession no. JNCG01000000) that was (erroneously) deposited in 2014 as *E. meningoseptica*. The paucity of our knowledge is an important challenge when dealing with *Elizabethkingia* infections and requires urgent attention from researchers and medical and health officials. *Elizabethkingia* spp. is still an understudied pathogen with an intrinsic multidrug resistance phenotype that has been reported in several countries around the world, causing opportunistic infections with high mortality rates. The apparent ubiquity of the pathogen, being found in diverse environments and animal hosts, and the presence of multiple antimicrobial resistance genes in its genome is a cause of serious concern as it could serve as a natural reservoir of antimicrobial resistance genes for horizontal transmission to other pathogenic microorganisms. Since currently, only whole-genome sequencing (and perhaps, MALDI-TOF MS in the near future) is able to accurately identify *Elizabethkingia* to the species level, cheaper but equally specific and sensitive identification or diagnostic methods need to be developed, as genome sequencing and MALDI-TOF MS are not available to most hospital diagnostic laboratories in poorer Southeast Asian countries. Nevertheless, a deeper understanding of the pathogen along with more precise diagnostic procedures, better accessibility of treatment options either with antibiotics or other alternatives such as bacteriophage therapy, and improvements in the prevention of transmission and infection will eventually enable better control of this extremely pathogenic and highly resistant bacteria.

## Figures and Tables

**Table 1:** Various sources of isolation of *Elizabethkingia* spp. in Southeast Asia.

Source of Isolation	Country of Origin	Citation
Blood	Malaysia, Singapore, Thailand, Indonesia, Cambodia	[8,13,15,23,24,25,26,36,65,91,92,93,94,95,96]
Peritoneal fluid	Malaysia	[91]
Cerebrospinal fluid (CSF)	Malaysia, Singapore	[15,23,91,97,98,99,100]
Contact lens	Malaysia	[101]
Hospital environment (aerators, sink drains and traps at ICUs, pediatric wards, surgical wards, orthopedic wards)	Singapore	[8,102,103,104]
Catheter tips	Singapore	[8]
Respiratory specimens	Singapore Malaysia	[8,65]
Rectal swabs	Singapore	[105]
Urine	Malaysia	[65]
Wound swabs	Malaysia	[65]
Nasal swabs	Malaysia	[65]
Vitreous culture	Singapore	[8]
Frogs (*Rana catesbeiana* (American bullfrogs) and *Theloderma bicolor* Chapa bug-eyed frogs, Warty toads (*Bombina microdeladigitora*), and Northern leopard frogs (*Lithobates pipiens*)	Malaysia Vietnam	[27,106,107]
Mosquitoes (*Anopheles minimus, Anopheles dirus, Anopheles maculatus, Anopheles sawadwongporni,* and *Anopheles dravidicus*)	Thailand	[90,108]
Fish (*Clarias gariepinus* (African sharptooth catfish) and *Pangasius hypophthalmus* (Tra catfish)	Malaysia, Vietnam	[28,75,109]
Retail sausages	Malaysia	[76]
*Gnetum gnemon* (Tree)	Malaysia	[110,111]

**Table 2:** *Elizabethkingia* spp. isolated from various Southeastern Asian countries based on the published reports until March 2022.

Isolate	Malaysia	Singapore	Thailand	Indonesia	Vietnam	Cambodia
	NR	NR	NR	NR	NR	NR
*E. meningoseptica* (CL)	12	11	2	4	-	1
*E. meningoseptica* (EN)	2	-	1	-	-	-
*E. anophelis* (CL)	1	2	-	-	-	2
*E. anophelis* (EN)	-	-	-	-	-	-
*E. miricola* (CL)	1	1	-	-	-	-
*E. miricola* (EN)	2	-	-	-	2	-
*Elizabethkingia* spp. (UI)	-	-	1	-	-	-

NR—number of published reports; CL—clinical isolates; EN—environmental isolate; UI—unidentified species.

## Data Availability

Not applicable.

## Supplementary Materials

The following supporting information can be downloaded at: https://www.mdpi.com/article/10.3390/microorganisms10050882/s1, Table S1: Genes encoding enzymes/proteins and efflux pumps involved in antibiotic resistance of the *Elizabethkingia* spp.; Table S2: Potential virulence-associated features among *Elizabethkingia* spp. as predicted using the Virulence Factor Database (VFDB) [39,40,41,43,44,46,59,60,61,62,72,73,77,119,120,121,122,123,124,125,126,127,128,129,130,131].
