# Supplementary material for: Epidemiology and Characteristics of Elizabethkingia spp. Infections in Southeast Asia
Source: Microorganisms. 2022 Apr 22;10(5):882. doi: 10.3390/microorganisms10050882 (PMC9144721; doi:10.3390/microorganisms10050882)
Supplement: Supplementary file 1 [file microorganisms-10-00882-s001.zip › microorganisms-1696780-supplementary.pdf]

Supplementary Table S1. Genes encoding enzymes/proteins and efflux pumps involved in Antibiotic Resistance of the *Elizabethkingia* spp.

**Antibiotic Class**	**Annotation***	***Gene***	***Elizabethkingia* spp.**	**Citation**
**Aminoglycosides**	Aminoglycoside Acetyltransferase (AAC3-I)	*aac3-i*	*E. meningoseptica*<br>*E. anophelis*<br>*E. miricola*	[1]
	Aminoglycoside 6-adenylyltransferase	*ant-6*	*E. meningoseptica*<br>*E. anophelis*<br>*E. miricola*	[2]
		ant-6-i	*E. anophelis*	[3]
	Aac(6'), aminoglycoside-6'-N-acetyltransferase	*aac6*	*E. meningoseptica*<br>*E. anophelis*	[4]
		aac6-lad	*E. anophelis*	[5]
**Aminosalicylate**	Thymidylate synthase (synthetase) catalyzes the conversion of dUMP to dTMP in the nucleic acid biosynthesis pathway.	*thyA*	*E. meningoseptica*<br>*E. anophelis*<br>*E. miricola*	[6]
**Beta-lactamases**	Metallo-beta-lactamase AIM-1. Imipenemase.	$bla_{\text{AIM-1}}$	*E. meningoseptica*<br>*E. anophelis*	[4]
	Metallo-beta-lactamase type 2	bla_{BlaB1}	*E. meningoseptica*<br>*E. anophelis*<br>*E. miricola*	[1,4,5,7–11]

		bla_{BlaB2}	*E. meningoseptica*	[6,7]
		bla_{BlaB3}	*E. meningoseptica*	[12]
		bla_{BlaB4}	*E. meningoseptica*	[12]
		bla_{BlaB5}	*E. meningoseptica*	[12]
		bla_{BlaB6}	*E. meningoseptica*	[7,11]
		bla_{BlaB9}	*E. meningoseptica*<br>*E. anophelis*<br>*E. miricola*	[7]
		bla_{BlaB10}	*E. meningoseptica*	[7]
		bla_{BlaB11}	*E.meningoseptica*	[7,8,13]
		bla_{BlaB12}	*E. meningoseptica*	[7]
		bla_{BlaB13}	*E. meningoseptica*	[6,7]
		bla_{BlaB14}	*E. anophelis*	[9]
		bla_{BlaB16}	*E. miricola*	[14]
		bla_{BlaB17}	*E.meningoseptica*	[12]
		bla_{BlaB19}	*E. miricola*	[14]
	Extended-spectrum β-lactamase.	$bla_{\text{SFO-1}}$	*E. meningoseptica*<br>*E. anophelis*	[4]

**Annotations are based on search against the Comprehensive Antibiotic Resistance Database (CARD: https://card.mcmaster.ca) and Universal Protein Resource (UniProt: https://www.uniprot.org/)

	Subclass B3 (metallo-) beta-lactamase hydrolize penicillins, cephalosporins and carbapenems / GOB beta-lactamase (subclass B3 (metallo-) beta-lactamase)	*bla*GOB-1	*E. meningoseptica*<br>*E. anophelis*<br>*E. miricola*	[1,6–9,12,13,15]
		*bla*GOB-4	*E. anophelis*	[3,5]
		*bla*GOB-8	*E. meningoseptica*	[7]
		*bla*GOB-9	*E. meningoseptica*	[7]
		*bla*GOB-10	*E. meningoseptica*	[7]
		*bla*GOB-11	*E. meningoseptica*	[7]
		*bla*GOB-12	*E. meningoseptica*	[7]
		*bla*GOB-13	*E. meningoseptica*<br>*E. anophelis*<br>*E. miricola*	[2,7]
		*bla*GOB-13b	*E. meningoseptica*	[7]
		*bla*GOB-14	*E. meningoseptica*	[7]
		*bla*GOB-15	*E. meningoseptica*	[7]
		*bla*GOB-16	*E. meningoseptica*	[7]
		*bla*GOB-17	*E. meningoseptica*	[7]
		*bla*GOB-18	*E. meningoseptica*	[8]
		*bla*GOB-19	*E. miricola*	[14]
	Hydrolyzed cephaloridine, cefotaxime, cephalothin, benzylpenicillin, and ceftazidime.	*TLA-1*	*E. meningoseptica*<br>*E. anophelis*<br>*E. miricola*	[5,6]
	Confers resistance to ceftazidime, cefotaxime and cefepime	*TLA-3*	*E. anophelis*	[16]

Supplementary Table S2. Potential virulence associated features among Elizabethkingia spp. as predicted using the Virulence Factor Database (VFDB).

Extended-spectrum β-lactamase enzyme. Chromosomal gene (blaACME) that encodes a class A β-lactamase conferring resistance to cephalosporins	*bla*$_{ACME-1}$	*E. meningoseptica*<br>*E. anophelis*<br>*E. miricola*	[2,3,5,8,9,12,15,17]
	bla$_{ACME-2}$	*E. meningoseptica*	[8,13]
ACC-3, AMPC cephalosporinase precursor protein ACC-3	*acc-3*	*E. meningoseptica*<br>*E. anophelis*	[4]
A serine beta-lactamase with a substrate specificity for cephalosporins.	*ampC*	*E. meningoseptica*	[13]
BlaIND-7, metallo-beta-lactamase IND-7	*IND-7*	*E. meningoseptica*<br>*E. anophelis*	[4]
Bla OCH-7, beta-lactams hydrolysis	*OCH-7*	*E. meningoseptica*<br>*E. anophelis*	[4]
Determinant of β-lactam resistance	*CPS-1*	*E. anophelis*	[16]
A subclass B3 metallo-beta lactamase resistant to carbapenems.	*ESP-1*	*E. anophelis*	[16]
	PEDO-1	*E. anophelis*	[16]
	PEDO-2	*E. anophelis*	[16]
	PEDO-3	*E. anophelis*	[16]
A subclass B3 LRA beta-lactamase conferring resistance to cephalosporin and penam	*LRA-17*	*E. anophelis*	[16]
	LRA-12	*E. anophelis*	[16]
Extended-spectrum beta-lactamase	*TEM-113*	*E. anophelis*	[16]
Extended-spectrum beta-lactamase PER-1 precursor	*per1_1*	*E. anophelis*	[10]
	per1_2	*E. anophelis*	[10]
Plasmid-mediated SPM metallo-beta-lactamase conferring resistance to carbapenem	*SPM-1*	*E. anophelis*	[16]
Beta-lactamase class-B	*bla*$_{LRA-12}$	*E. anophelis*	[16]

		bla_{LRA-19}	*E. anophelis*	[16]
	SubclassB1. Hydrolyze a variety of beta-lactams, including penicillins, cephalosporins, and carbapenems.	*SMB-1*	*E. meningoseptica*<br>*E. anophelis*<br>*E. miricola*	[1]
**Chloramphenicol**	Chloramphenicol Acetyltransferase gene	*cat*	*E. meningoseptica*<br>*E. anophelis*<br>*E. miricola*	[1,18]
	Plasmid-encoded variant of the cat gene	*catB*	*E. anophelis*	[3]
		catB2	*E. meningoseptica*<br>*E. anophelis*<br>*E. miricola*	[6,10,16,19]
		catB3	*E. anophelis*	[16]
		catB4	*E. anophelis*	[5]
		catB6	*E. anophelis*	[16]
		catB7	*E. anophelis*	[16]
		catB8	*E. anophelis*	[16]
		catB9	*E. anophelis*	[16]
		catB10	*E. anophelis*	[16]

VFID	VF_Category	VF_Name	Gene	Annotation	Bacteria	Reference

	Confers sulfonamide (sulfathiazole) and bicyclomycin resistance	*bcr*	*E. meningoseptica*<br>*E. anophelis*<br>*E. miricola*	[2]
**Efflux Pump**	A cytoplasmic membrane component of the CeoAB-OpcM efflux pump	*ceoB*	*E. meningoseptica*<br>*E. anophelis*<br>*E. miricola*	[6]
	MdsB is the inner membrane transporter of the multidrug and metal efflux complex MdsABC	*mdsB*	*E. meningoseptica*<br>*E. anophelis*<br>*E. miricola*	[6]
	ABC efflux pump gene, confers resistance to pleuromutilin antibiotics.	*taeA*	*E. meningoseptica*<br>*E. anophelis*<br>*E. miricola*	[6]
	Inner membrane transporter of the AdeFGH multidrug efflux complex.	*adeG-1*	*E. meningoseptica*<br>*E. anophelis*<br>*E. miricola*	[6]
		adeG-2	*E. meningoseptica*<br>*E. anophelis*<br>*E. miricola*	[6]
	Acell division regulator protein that is also a positive regulator of AcrAB only when it's expressed from a plasmid.	*sdiA*	*E. meningoseptica*<br>*E. anophelis*<br>*E. miricola*	[6]
	An AraC-family regulator that promotes mdtEF expression	*gadW*	*E. meningoseptica*<br>*E. anophelis*<br>*E. miricola*	[6]
	A response regulator that binds to the norA promoter to activate expression. ArlR must first be phosphorylated by ArlS.	*arlR*	*E. meningoseptica*<br>*E. anophelis*<br>*E. miricola*	[6]

	A repressor for the CmeABC multidrug efflux pump, binding to the cmeABC promoter region	*cmeR*	*E. meningoseptica*<br>*E. anophelis*<br>*E. miricola*	[6]
	An ATP-binding cassette (ABC) transporter that exports macrolides with 14- or 15- membered lactones.	*macB*	*E. meningoseptica*<br>*E. anophelis*<br>*E. miricola*	[1]
	Efflux pump membrane transporter BepE	*bepE_1*	*E. anophelis*	[10]
		bepE_2	*E. anophelis*	[10]
		bepE_4	*E. anophelis*	[10]
		bepE_5	*E. anophelis*	[10]
		bepE_6	*E. anophelis*	[10]
		bepE_7	*E. anophelis*	[10]
	Inner membrane transporter the CmeABC multidrug efflux complex	*cmeB*	*E. meningoseptica*<br>*E. miricola*<br>*E. anophelis*<br>*E. bruuniana*	[20]
	Protein subunit of AcrA-AcrB-TolC multidrug efflux complex. AcrB functions as a herterotrimer which forms the inner membrane component and is primarily responsible for substrate recognition and energy transduction by acting as a drug/proton antiporter	*acrB*	*E. meningoseptica*<br>*E. miricola*<br>*E. anophelis*<br>*E. bruuniana*<br>*E. ursingii*<br>*E. occulta*	[20]
	Subunit of the qac multidrug efflux pump	*qacH*	*E. anophelis*	[16]
	Efflux pump of the SMR family of transporters	*abeS*	*E. anophelis*	[6,16]
**Elfamycin**	Involved in the lipid A biosynthesis	*lpxC*	*E. anophelis*	[16]
**Fluoroquinolones**	OqxA membrane-fusion protein. component of RND-type multidrug efflux pump that confers resistance to olaquindox	*oqxA*	*E. meningoseptica*<br>*E. anophelis*	[4]

	OqxB integral membrane protein. component of RND-type multidrug efflux pump that confers resistance to olaquindox	*oqxBgb*	*E. meningoseptica*<br>*E. anophelis*	[4]
	A30S ribosomal protein S10 involved in the binding of tRNA to the ribosomes	*rpsJ*	*E. anophelis*	[16]
	Multidrug resistance protein MexA precursor	*mexA_3*	*E. anophelis*	[10]
	Multidrug resistance protein MdtE precursor	*mdtE_1*	*E. anophelis*	[10]
	Membrane fusion protein of the MexEF-OprN multidrug efflux complex	*mexE*	*E. meningoseptica*<br>*E. anophelis*<br>*E. miricola*	[1]
	Membrane fusion protein of the efflux complex MexGHI-OpmD	*mexH*	*E. meningoseptica*<br>*E. anophelis*<br>*E. miricola*	[1,10]
	Lipid A export ATP-binding/permease protein MsbA	*msbA*	*E. anophelis*	[10]
**GCN5-related N-acetyltransferases**	Catalyzes the transfer of an acetyl group from acetyl coenzyme A (AcCoA) to an acceptor substrate and releases both CoA and the acetylated product	*GNAT*	*E. meningoseptica*<br>*E. anophelis*<br>*E. miricola*	[1]
**Glycopeptides**	D-specific alpha-ketoacid dehydrogenase that synthesizes D-lactate. D-lactate is incorporated into the end of the peptidoglycan subunits, decreasing vancomycin binding affinity.	*vanH*	*E. meningoseptica*<br>*E. anophelis*	[4]
	vanRA, also known as vanR, is a vanR variant found in the vanA gene cluster	*vanRA*	*E. meningoseptica*<br>*E. anophelis*	[4]
	vanRB is a vanR variant found in the vanB gene cluster	*vanRB*	*E. meningoseptica*<br>*E. anophelis*	[4]
	vanRF is a vanR variant found in the vanF gene cluster	*vanRF*	*E. meningoseptica*<br>*E. anophelis*<br>*E. miricola*	[6]

VFG0864	Adherence	AggR	*aggR*	Putative transcriptional activator aggR (AAF-III) regulatory protein	*E. anophelis*	[25]

	vanXYL is a vanXY variant found in the vanL gene cluster	*vanXYL*	*E. meningoseptica*<br>*E. anophelis*<br>*E. miricola*	[6]
	Serine racemase VanT. Converts L-serine to D-serine; involved in vancomycin resistance	*vanT*	*E. meningoseptica*<br>*E. anophelis*	[4]
	vanWB, also known as vanW, is a vanW variant found in the vanB gene cluster	*vanWB*	*E. meningoseptica*<br>*E. anophelis*	[4]
	VanC-type vancomycin resistance DNA-binding response regulator VanR	*vanRc3*	*E. meningoseptica*<br>*E. anophelis*	[4]
	vanRM is a vanR variant found in the vanM gene cluster	*vanRM*	*E. meningoseptica*<br>*E. anophelis*	[4]
	VanB is a D-Ala-D-Ala ligase homolog similar to VanA, and can synthesize D-Ala-D-Lac, an alternative substrate for peptidoglycan synthesis that reduces vancomycin binding affinity	*vanB*	*E. meningoseptica*	[21]
	vanW is a vancomycin resistance gene.	*vanW*	*E. meningoseptica*<br>*E. miricola*<br>*E. anophelis*<br>*E. bruuniana*<br>*E. ursingii*<br>*E. occulta*	[20,21]
**Isoniazid**	A catalase-peroxidase that catalyzes the activation of isoniazid	*katG*	*E. meningoseptica*<br>*E. anophelis*<br>*E. miricola*	[6]
**Macrolide-lincosamide-streptogramin**	ABC-F subfamily protein involved in carbomycin resistance.	*carA*	*E. meningoseptica*<br>*E. anophelis*	[4,5]
	lmrA is the repressor to the lmrAB and lincomycin resistant	*lmrA*	*E. meningoseptica*<br>*E. anophelis*	[4]

	Macrolide efflux pump gene located in the vicinity of sul3.	*mefB*	*E. meningoseptica*<br>*E. anophelis*	[4]
	ABC-F subfamily protein that confers resistance to erythromycin and streptogramin B antibiotics. It is associated with plasmid DNA.	*msrB1*	*E. meningoseptica*<br>*E. anophelis*<br>*E. miricola*	[6]
		msrB2	*E. meningoseptica*<br>*E. anophelis*<br>*E. miricola*	[6]
	ABC-F subfamily protein that confers resistance to erythromycin and streptogramin B antibiotics. It is associated with plasmid DNA.	*msrE*	*E. meningoseptica*<br>*E. anophelis*	[4,5]
	ATP-binding protein. oleandomycin resistance and secretion	*oleB*	*E. meningoseptica*<br>*E. anophelis*	[4,5]
	Tylosin resistance protein (tlrC) gene	*tlrC*	*E. meningoseptica*<br>*E. anophelis*	[4,5]
	Streptogramin A acetyl transferase (sat) gene. Confers resistance to class A streptogramins	*vatF*	*E. meningoseptica*<br>*E. anophelis*	[4]
	Plasmid-mediated acetyltransferase streptogramin resistant genes.	*vatA*	*E. meningoseptica*<br>*E. anophelis*<br>*E. miricola*	[6]
		vatB	*E. anophelis*	[5]
		vatH	*E. meningoseptica*	[6]
	ErmF confers the MLSb phenotype.	*ermF*	*E. anophelis*	[16]
	ABC-F subfamily protein that confers resistance to streptogramin A antibiotics and related compounds. It is associated with plasmid DNA.	*vgaALC*	*E. meningoseptica*<br>*E. anophelis*	[4]

VFG1206	Antigen 85	*fbpC*	Secreted antigen 85-C FbpC (85C) (antigen 85 complex C) (AG58C)	*E. anophelis*	[25]

	ErmB confers the MLSb phenotype. Similar to ErmC, expression of ErmB is inducible by erythromycin. The leader peptide causes attenuation of the mRNA and stabilizes the structure preventing further translation. When erythromycin is present, it binds the leader peptide causing a change in conformation allowing for the expression of ErmB.	*ermB*	*E. meningoseptica*<br>*E. anophelis*<br>*E. miricola*	[6]
	Macrolide resistance, part of ABC transporter complex LolCDE.	*lolD*	*E. meningoseptica*<br>*E. anophelis*<br>*E. miricola*	[2]
	A translocase in the emrB -TolC efflux protein that confers resistance to erythromycin.	*emrB*	*E. meningoseptica*<br>*E. anophelis*<br>*E. miricola*	[2]
	Cfr, rRNA methylase, mediates the PhLOPSA resistance phenotype	*cfrA*	*E. meningoseptica*<br>*E. anophelis*	[4]
**Multiple antibiotic resistance**	Multiple antibiotic resistance MAR locus	*marA*	*E. miricola*<br>*E. bruuniana*	[20]
		marB	*E. miricola*<br>*E. bruuniana*	[20]
		marC	*E. ursingii*<br>*E. occulta*	[20]
**Peptide**	bacA gene product (BacA) recycles undecaprenyl pyrophosphate during cell wall biosynthesis which confers resistance to bacitracin.	*bacA*	*E. meningoseptica*<br>*E. anophelis*<br>*E. miricola*	[6]
**Quinolone**	Encoding the DNA gyrase A subunit (gyrA). Resistance to fluoroquinolones–DNA gyrase	*gyrA*	*E. meningoseptica*<br>*E. miricola*<br>*E. anophelis*<br>*E. bruuniana*<br>*E. ursingii*<br>*E. occulta*	[2,20,22,23]

	Essential for DNA supercoiling. Confers antibiotic resistance by preventing drugs from binding the beta-subunit of gyrase.	*gyrB*	*E. meningosepticaa*<br>*E. miricola*<br>*E. anophelis*<br>*E. bruuniana*<br>*E. ursingii*<br>*E. occulta*	[2,6,20,22]
	DNA topoisomerase IV subunit B.	*parC*	*E. meningoseptica*<br>*E. miricola*<br>*E. anophelis*<br>*E. bruuniana*<br>*E. ursingii*<br>*E. occulta*	[20]
		parE	*E. meningoseptica*<br>*E. miricola*<br>*E. anophelis*<br>*E. bruuniana*<br>*E. ursingii*<br>*E. occulta*	[2]<br>Liang et al., 2019
**Rifampicin**	A chromosome-encoded ribosyltransferase	*arr-1*	*E. anophelis*	[16]
	ADP-ribosyltransferase. Resistance to rifampin.	*arr7*	*E. meningoseptica*<br>*E. anophelis*	[4]
**Rifamycin**	RNA polymerase beta subunit. Resistance to the salinamide A, salinamide B, rifampicin, streptolydigin, CBR703, myxopyronin, and lipiarmycin due to mutations.	*rpoB*	*E. meningoseptica*<br>*E. anophelis*<br>*E. miricola*	[6]
**Sulfonamide**	A sulfonamide resistant dihydropteroate synthase, linked to class 1 integrons.	*sul1*	*E. meningoseptica*	[24]
		sul2	*E. meningoseptica*	[24]
	Dihydrofolate reductase DHFR	*dhfR*	*E. meningoseptica*<br>*E. anophelis*<br>*E. miricola*	[2]

	Dihydropteroate synthase, prevents sulfonamide antibiotics from inhibiting its role in folate synthesis.	*folP*	*E. meningoseptica*<br>*E. anophelis*<br>*E. miricola*	[2]
**Tetracycline**	An oxytetracycline resistance ribosomal protection protein	*otrA*	*E. meningoseptica*<br>*E. anophelis*	[4–6]
	Tetracycline efflux protein gene	*otrB*	*E. meningoseptica*<br>*E. anophelis*	[4]
	Tetracycline efflux protein, class C	*tetA*	*E. anophelis*	[2,10]
	A tetracycline efflux protein. It confers resistance to tetracycline, doxycycline, and minocycline, but not tigecycline.	*tetB*	*E. meningoseptica*<br>*E. anophelis*<br>*E. miricola*	[6,16]
	A flavin-dependent monooxygenase that hydroxylates position 11a of the tetraketide group thus confering resistance to antibiotics	*tetX*	*E. meningoseptica*<br>*E. anophelis*	[1,4,5,9,16]
	Terminates tetracyclin's inhibitory effect on protein synthesis by ribosomal non-covalent modification	*tetO*	*E. meningoseptica*<br>*E. anophelis*<br>*E. miricola*	[6]
	A tetracycline ribosomal protection protein found on the same operon as tetA(P), a tetracycline efflux protein	*tetB(P)*	*E. meningoseptica*<br>*E. anophelis*<br>*E. miricola*	[6]
**Trimethoprim**	A chromosome-encoded dihydrofolate reductase. Determinant of diaminopyrimidine resistance	*dfrE*	*E. anophelis*	[16]
		dfrA3	*E. meningoseptica*<br>*E. anophelis*<br>*E. miricola*	[6]
	An integron-encoded dihydrofolate reductase	*dfrA12*	*E. meningoseptica*	[24]
		dfrA20	*E. anophelis*	[5]

DHFRXVI, trimethoprim resistant dihydrofolate reductase	*dfr16*	*E. meningoseptica*<br>*E. anophelis*	[4]

			(mycolyl transferase 85C)<br>(fibronectin-binding protein C)		
VFG1797	C5a peptidase	*scpB*	Streptococcal C5a peptidase	*E. anophelis*	[25]
VFG1931	Campylobacter adhesion to fibronectin (CadF)	*cadF*	Outer membrane fibronectin-binding protein	*E. anophelis*<br>*E. miricola*	[1,5,10,25]
VFG1354	Choline binding proteins (CBPs)	*cbpE*	Choline binding protein E	*E. anophelis*	[5,25]
VFG001354		*pce*	Choline binding protein E	*E. meningoseptica*<br>*E. anophelis*<br>*E. miricola*	[1,10]
*WP_069214179	Curli fibers	*CurEm1*	Hypothetical protein	*E. meningoseptica*	[6]
*WP_069214180		*CurEm2*	Hypothetical protein	*E. meningoseptica*	[6]
*WP_069214181		*CurEm3*	Curli assembly protein CsgF	*E. meningoseptica*	[6]
*WP_070904486		*CurEm4*	Curli production assembly protein CsgG	*E. meningoseptica*	[6]
VFG000457		*csgB*	Minor curlin subunit precursor, curli nucleator protein	*E. meningoseptica*	[1]

VFG000462			*csgG*	Curli production assembly/transport protein	*E. meningoseptica*	[1]
VFG004125			*csgD*	DNA-binding transcriptional regulator	*E. meningoseptica*<br>*E. anophelis*<br>*E. miricola*	[1,10]
VFG010763		EF-Tu	*tuf*	Translation elongation factor	*E. meningoseptica*<br>*E. anophelis*<br>*E. miricola*<br>*E. bruuniana*<br>*E. ursingii*<br>*E. occulta*	[16,20,26]
VFG001855		Hsp60	*htpB*	60K heat shock protein	*E. meningoseptica*<br>*E. anophelis*<br>*E. miricola*<br>*E. bruuniana*<br>*E. ursingii*<br>*E. occulta*	[1,3,5,10,16,20,25–27]
VFG045346		Immunogenic lipoprotein A	*IlpA*	Immunogenic lipoprotein A	*E. meningoseptica*<br>*E. anophelis*<br>*E. miricola*	[1,10,16]
VFG000335V<br>FG002008<br>VFG002064		P5 protein	*ompP5*	Outer membrane protein P5 (ompA), human factor H binding protein	*E. anophelis*	[10]
VFG0101		Toxin-coregulated pilus (TCP)	*toxT*	TCP pilus virulence regulatory protein	*E. meningoseptica*<br>*E. anophelis*<br>*E. miricola*	[1,25]
VFG0872		Type I pili	*fimE*	Type 1 fimbriae Regulatory protein fimE	*E. anophelis*	[25]
VFG1234		Type IV pili	*chpD*	Probable transcriptional regulator	*E. anophelis*	[25]

VFG1226			*pilH*	Pilin-like protein may involving in pseudopilus formation	*E. anophelis*	[25]
VFG1214			*pilR*	Two-component response regulator	*E. meningoseptica*<br>*E. anophelis*<br>*E. miricola*	[1,10,25,27]
VFG1225			*pilG*	Twitching motility protein	*E. miricola*	[1,25]
SCV48	Antimicrobial activity/Compe titive advantage	Macrophage-inducible gene-5 (Mig-5)	*mig-5*	Carbonic anhydrase chloroplast precursor	*E. meningoseptica*	[20]
	Biofilm	AdeFGH efflux pump	*adeG*	Cation/multidrug efflux pump	*E. meningoseptica*<br>*E. anophelis*<br>*E. miricola*<br>*E. bruuniana*<br>*E. ursingii*<br>*E. occulta*	[20,26]
VFG0122		Alginate (Mucoid exopolysaccharide)	*algD*	GDP-mannose 6-dehydrogenase AlgD	*E. anophelis*	[25]
VFG0130			*algI*	Alginate o-acetyltransferase AlgI	*E. anophelis*	[25]
VFG0119			*algR*	Alginate biosynthesis regulatory protein AlgR	*E. anophelis*	[25]
VFG0120			*algZ*	Sigma factor AlgU	*E. anophelis*	[25]
VFG001284		Intercellular adhesion proteins	*icaR*	Ica operon transcriptional regulator	*E. meningoseptica*<br>*E. anophelis*<br>*E. miricola*	[1,10]

VFG0152		Quorum sensing	*rhlR*	Transcriptional regulator RhlR	*E. anophelis*	[5,25]
			bspR2	N-acyl-homoserine lactone dependent regulatory protein	*E. anophelis*	[10]
VFG2538			*pmlR/bspR1*	N-acylhomoserine lactone dependent regulatory protein	*E. meningoseptica*<br>*E. anophelis*<br>*E. miricola*	[1,10,25]
VFG1417	Cellular metabolism	PanC/PanD	*panC*	Pantoate--beta-alanine ligase	*E. anophelis*	[5,25,27]
VFG1416			*panD*	Aspartate 1-decarboxylase precursor	*E. meningoseptica*<br>*E. anophelis*<br>*E. miricola*<br>*E. bruuniana*<br>*E. ursingii*<br>*E. occulta*	[5,20,25,27]
VFG1381		Isocitrate lyase	*aceA*	Isocitrate lyase aceA	*E. anophelis*	[5,10,27]
VFG2445	Effector delivery system	Bsa T3SS	*bapB*	Acyl carrier protein	*E. anophelis*	[25]
VFG2440			*bprB*	Two-component response regulator	*E. anophelis*	[25]
VFG2464			*bsaN*	AraC family transcriptional regulator, regulates basal T6SS1 expression	*E. anophelis*	[25]
VFG2110		Dot/Icm T4SS secreted effectors	*vipD*	Dot/Icm type IV secretion system effector VipD, Phospholipase A1	*E. anophelis*	[5,25]

VFG041304			*lirB*	Dot/Icm type IV secretion system effector	*E. meningoseptica*<br>*E. anophelis*<br>*E. miricola*	[1,10]
VFG010763			*vpdB*	Dot/Icm type IV secretion system effector	*E. meningoseptica*<br>*E. anophelis*<br>*E. miricola*	[1,10]
VFG002064		Hcp1 secretion island I (HSI-I)	*dotU1*	Type VI secretion system protein DotU	*E. meningoseptica*<br>*E. anophelis*<br>*E. miricola*	[1]
VFG002059			*tagT*	Type six secretion associated protein TagT, ATP-binding component of ABC transporter	*E. meningoseptica*<br>*E. anophelis*<br>*E. miricola*	[1,10]
VFG2059			*PA0073*	Type six secretion associated protein TagT, ATP-binding component of ABC transporter	*E. anophelis*	[5,25]
VFG2064			*PA0078*	Type VI secretion system protein DotU	*E. anophelis*	[5,25]
VFG045340		Rab2 interacting conserved protein A	*ricA*	Rab2 interacting conserved protein A	*E. meningoseptica*	[1]
VFG0208		T3SS	*exsA*	Type III secretion system transcriptional regulator	*E. anophelis*	[25]

VFG2085		T6SS	*vasH*	Type VI secretion system regulatory protein	*E. meningoseptica*<br>*E. anophelis*<br>*E. miricola*	[1,5,25]
VFG2480		T6SS-1	*clpV*	Clp-type ATPase chaperone protein	*E. meningoseptica*<br>*E. anophelis*<br>*E. miricola*	[1,5,10,25]
VFG1746		TTSS (chromosomally encoded)	*YPO0255*	Putative two-component response regulator	*E. anophelis*	[5,25]
VFG1747			*YPO0256*	Two-component sensor/regulator	*E. anophelis*	[25]
VFG1751			*YPO0260*	Putative AraC-family regulatory protein	*E. anophelis*	[25]
VFG0555		TTSS (SPI-1 encode)	*invC*	Type III secretion system ATPase SpaI/InvC	*E. anophelis*	[25]
VFG0492		TTSS (SPI-2 encode)	*ssrB*	Secretion system regulator: transcriptonal activator, homologous with degU / uvrY / bvgA	*E. anophelis*	[25]
VFG1794		TTSS (Ysa)	*ysrS*	Sensor kinase protein	*E. anophelis*	[25]
		TTSS secreted effectors	*exoU*	Type III secretion system effector ExoU, phospholipase A2 activity	*E. anophelis*	[10]
VFG2276	Exoenzyme	kappa-toxin	*colA*	Collagenase	*E. meningoseptica*<br>*E. anophelis*<br>*E. miricola*	[1,5,25]

VFG2279		mu-toxin	*nagJ*	Hyaluronidase	*E. anophelis*	[25]
VFG2281			*nagL*	Hyaluronidase	*E. anophelis*	[25]
VFG2284		Sialidase	*nanJ*	Exo-alpha-sialidase	*E. anophelis*	[25]
VFG2283			*nanI*	exo-alpha-sialidase	*E. meningoseptica*<br>*E. miricola*	[1]
VFG0674		SMase	*smcL*	Sphingomyelinase-c	*E. miricola*	[1]
VFG005767	Exotoxin	Beta-hemolysin/cytolysin	*cylG*	3-ketoacyl-ACP-reductase	*E. meningoseptica*<br>*E. anophelis*<br>*E. miricola*	[1,10]
VFG1269		Invasive Adenylate cyclase / haemolysin	*cyaB*	Cyclolysin secretion ATP-binding protein	*E. anophelis*	[25]
VFG000843		Hemolysin	*hlyD*	Hemolysin transport protein	*E. meningoseptica*<br>*E. anophelis*<br>*E. miricola*	[1]
VFG0841<br>VFG1557<br>VFG000907			*hlyB*	Hemolysin B	*E. miricola*<br>*E. anophelis*	[1,25]
VFG0073		PlcA	*plcA*	Phospholipase C	*E. meningoseptica*<br>*E. anophelis*	[25,26]
VFG2274		Phospholipase C	*plc*	Phospholipase C	*E. meningoseptica*<br>*E. anophelis*	[20]

					E. miricola<br>*E. bruuniana*<br>*E. ursingii*<br>*E. occulta*	
VFG1394		Phospholipase D	*plcD*	Phosphatidylserine/phosphatidyl glycerophosphate/cardiolipin synthase	*E. anophelis*	[25]
			Pld	Phospholipase D	*E. meningoseptica*<br>*E. anophelis*	[26]
VFG0028	Immune modulation	Brk	*brkB*	Serum resistance protein	*E. anophelis*	[25]
		Capsule	*cap4D*	Tyrosine-protein kinase	*E. meningoseptica*<br>*E. anophelis*<br>*E. miricola*	[6]
VFG1300			*cap8D*	Capsular polysaccharide synthesis enzyme Cap8D	*E. anophelis*	[5,25]
VFG1301			*cap8E*	Capsular polysaccharide synthesis enzyme	*E. anophelis*	[1,6,25]
VFG1303			*cap8G*	Capsular polysaccharide synthesis enzyme	*E. anophelis*	[1,6]
			capE	CapE, involved in Poly-gamma-glutamate synthesis	*E. anophelis*	[3]
			capG	Capsular polysaccharide synthesis enzyme CapG	*E. anophelis*	[3]

		capL	Capsular polysaccharide synthesis enzyme	*E. anophelis*	[16]
VFG1989		*Cj1416c*	Sugar nucleotidyltransferase	*E. anophelis*	[1]
VFG1958		*Cj1430c*	Putative aminotransferase	*E. anophelis*	[25]
VFG1965		*Cj1437c*	Aminotransferase	*E. anophelis*	[25]
VFG1372		*cps4H*	Capsular polysaccharide biosynthesis protein Cps4H	*E. anophelis*	[25]
VFG1348		*cpsE*	Glycosyl transferase, group 2 family protein	*E. anophelis*	[25]
VFG1344		*cpsM*	Polysaccharide biosynthesis protein CpsM(V)	*E. anophelis*	[25]
VFG1338		*neuB*	N-acetyl neuramic acid synthetase	*E. anophelis*	[1]
		rmlA	Glucose-1-phosphate thymidylyltransferase	*E. anophelis*	[16]
		ugd	UDP-glucose 6-dehydrogenase	*E. anophelis*	[16]
VFG1302		*cap8F*	Capsular polysaccharide synthesis enzyme Cap8F	*E. meningoseptica* *E. miricola*	[6]
VFG1311		*cap8O*	Capsular polysaccharide synthesis enzyme Cap8O	*E. meningoseptica*	[6]

VFG1368		*cps4D*	Capsular polysaccharide biosynthesis protein Cps4D	*E. meningoseptica*<br>*E. miricola*	[6]
VFG0696		*bexA*	ATP-dependent polysaccharide export protein BexA	*E. meningoseptica*<br>*E. anophelis*<br>*E. miricola*	[1,5,10,25]
		cap4F	Capsular polysaccharide synthesis enzyme	*E. meningoseptica*<br>*E. anophelis*<br>*E. miricola*	[1,6]
VFG1306		*cap8J*	Capsular polysaccharide synthesis enzyme	*E. meningoseptica*<br>*E. anophelis*<br>*E. miricola*	[1,5,10,25]
VFG0679		*capD*	Capsular polysaccharide synthesis enzyme CapD	*E. meningoseptica*<br>*E. anophelis*<br>*E. miricola*	[6,25]
VFG1369		*cps4E*	Capsular polysaccharide biosynthesis protein	*E. meningoseptica*<br>*E. anophelis*	[1,27]
VFG1352		*cpsA*	Undecaprenyl diphosphate synthase	*E. meningoseptica*<br>*E. anophelis*<br>*E. miricola*	[1]
VFG1351		*cpsB*	Phosphatidate cytidylyltransferase	*E. meningoseptica*<br>*E. anophelis*<br>*E. miricola*	[1]
VFG1349		*cpsD*	Autokinase	*E. meningoseptica*<br>*E. anophelis*<br>*E. miricola*	[1,25]

VFG1341			*cpsJ*	Glycosyl transferase CpsJ(V)	*E. meningoseptica*<br>*E. anophelis*<br>*E. miricola*	[1,10,25]
VFG1342			*cpsO*	Glycosyl transferase CpsO(V)	*E. meningoseptica*<br>*E. anophelis*<br>*E. miricola*	[1,5,10,25]
VFG2364			*fcl*	GDP-L-fucose synthetase	*E. meningoseptica*<br>*E. anophelis*<br>*E. miricola*<br>*E. ursingii*<br>*E. occulta*	[1,5,10,16,20,25,26]
VFG1971			*kpsF*	D-arabinose 5-phosphate isomerase	*E. meningoseptica*<br>*E. anophelis*<br>*E. miricola*	[1,5,10,25,27]
VFG1449			*kpsT*	Capsular polysaccharide ABC transporter, ATP-binding protein	*E. meningoseptica*<br>*E. anophelis*<br>*E. miricola*	[1,5,10,25]
			pgi	Glucose-6-phosphate isomerase	*E. meningoseptica E. anophelis*<br>*E. miricola*<br>*E. ursingii*<br>*E. occulta*	[16,20,26]
VFG001968			*Cj1440c*	Sugar transferase	*E. miricola*	[1,25]
VFG2363		Capsule I	*manC*	Mannose-1-phosphate guanylyl transferase	*E. anophelis*	[25]

VFG2550		*wcbP*	Capsular polysaccharide biosynthesis dehydrogenase / reductase	*E. anophelis*	[25]
VFG2546		*wcbT*	Acyl-CoA transferase	*E. anophelis*	[25]
VFG002563		*wzt2*	ATP-binding ABC transporter capsular polysaccharide export protein	*E. meningoseptica*<br>*E. anophelis*<br>*E. miricola*	[1,5,10,25]
VFG0307	HP-NAP	*napA*	Neutrophil activating protein NapA	*E. anophelis*	[25]
VFG000964	Hyaluronic acid capsule	*hasC*	UDP-glucose pyrophosphorylase	*E. anophelis*	[1]
VFG1936	Lipooligosacchari de (LOS)	*Cj1135*	Glucosyltransferase	*E. anophelis*	[1,25]
VFG1939		*Cj1138*	Glycosyltransferase	*E. anophelis*	[1,25]
VFG013354		*kfiC*	Lipopolysaccharide biosynthesis protein	*E. anophelis*<br>*E. miricola*	[1]
VFG0328		*lic1C*	ic-1 operon protein (licC)	*E. anophelis*	[25]
VFG013487		*lsgE*	Glycosyltransferase	*E. anophelis*	[1]
VFG013248		*msbA*	Lipid transporter ATP-binding/permease	*E. anophelis*	[1,10]
VFG013265		*orfM*	Deoxyribonucleotide triphosphate pyrophosphatase	*E. anophelis*	[1]

VFG2027		*waaE*	Putative ADP-heptose synthase	*E. anophelis*	[25]
VFG1947		*waaV*	Lipooligosaccharide biosynthesis glycosyltransferase	*E. anophelis*	[25]
VFG1937		*Cj1136*	Glucosyltransferase	*E. meningoseptica*<br>*E. anophelis*	[1,10,25]
VFG1938		*Cj1137c*	Glycosyltransferase	*E. meningoseptica*<br>*E. anophelis*<br>*E. miricola*	[6,25]
VFG0079		*clpC*	Endopeptidase Clp ATP-binding chain C	*E. meningoseptica*<br>*E. anophelis*<br>*E. miricola*	[1,5,10,25,27]
VFG013465		*kdsA*	2-dehydro-3-deoxyphosphooctonate aldolase	*E. meningoseptica*<br>*E. anophelis*<br>*E. miricola*	[1,10]
VFG013471		*lgtA*	N-acetylglucosamine glycosyltransferase	*E. meningoseptica*<br>*E. anophelis*<br>*E. miricola*	[1]
VFG013390		*lpxA*	UDP-N-acetylglucosamine acyltransferase	*E. meningoseptica*<br>*E. anophelis*<br>*E. miricola*	[1]
VFG013265		*orfM*	Deoxyribonucleotide triphosphate pyrophosphatase	*E. meningoseptica*<br>*E. anophelis*<br>*E. miricola*	[1,10]
VFG013368		*rffG*	dTDP-glucose 46-dehydratase	*E. meningoseptica*<br>*E. anophelis*	[1,10]

				E. miricola	
VFG0037	Lipopolysaccharide (LPS)	*bplB*	probable acetyltransferase	*E. anophelis* *E. miricola*	[1,5,10,25,27]
VFG0033		*bplF*	Lipopolysaccharide biosynthesis protein	*E. anophelis*	[25]
VFG0032		*bplG*	Probable sugar transferase	*E. anophelis* *E. miricola*	[1,5,10,16,25]
VFG0314		*gluE*	UDP-glucose 4-epimerase	*E. anophelis* *E. miricola*	[1,5,10,25]
VFG0313		*gluP*	Glucose/galactose transporter	*E. anophelis*	[25]
VFG0670		*gtrB*	Bactoprenol glucosyl transferase	*E. anophelis*	[25,27]
VFG0323		*lpxB*	Lipid-A-disaccharide synthase	*E. anophelis*	[25]
VFG2226		*per*	Perosamine synthetase	*E. anophelis*	[25]
VFG0321		*rfbD*	GDP-D-mannose dehydratase	*E. anophelis*	[25]
VFG0315		*rfbM*	Mannose-6-phosphate isomerase	*E. anophelis*	[1,25]
VFG2230		*wbkC*	GDP-mannose 4,6-dehydratase / GDP-4-amino-4,6-dideoxy-D-mannose formyltransferase	*E. anophelis*	[25]
VFG2228		*wzt*	O-antigen export system ATP-binding protein	*E. anophelis*	[5,25]

VFG011430			*acpXL*	Acyl carrier protein	*E. meningoseptica*<br>*E. anophelis*<br>*E. miricola*<br>*E. ursingii*<br>*E. occulta*	[1,20]
VFG0036			*bplC*	Lipopolysaccharide biosynthesis protein	*E. meningoseptica*<br>*E. anophelis*<br>*E. miricola*	[1,5,10,25,27]
VFG0320			*kdtB*	Lipopolysaccharide core biosynthesis protein	*E. meningoseptica*<br>*E. anophelis*<br>*E. miricola*	[1,5,10,25,27]
			wbtI	DegT/DnrJ/EryC1/StrS family aminotransferase	*E. meningoseptica*<br>*E. anophelis*<br>*E. occulta*	[20,26]
VFG2361		O-antigen	*galE*	UDP-glucose 4-epimerase	*E. meningoseptica*<br>*E. anophelis*<br>*E. miricola*<br>*E. ursingii*<br>*E. occulta*	[1,5,10,16,20,25–27]
VFG2376			*ddhB*	CDP-glucose 4,6-dehydratase	*E. anophelis*	[25]
VFG2225			*gmd*	GDP-mannose 4,6-dehydratase	*E. anophelis*<br>*E. miricola*	[1,5,10,16,25]
VFG2374			*prt*	Paratose synthase	*E. anophelis*<br>*E. miricola*	[1,25]
VFG2366			*rfpB*	Putative glycosyltransferase	*E. anophelis*	[25]

VFG2373			*wbcC*	Putative glycosyltransferase	*E. anophelis*	[25]
VFG2368			*wbcG*	Putative glycosyltransferase	*E. anophelis*	[25]
VFG1443		OmpA	*ompA*	outer membrane protein OmpA	*E. anophelis*	[25]
VFG1408		PDIM (Phthiocerol dimycocerosate)	*mas*	Putative multifunctional mycocerosic acid synthase membrane-associated MAS	*E. anophelis*	[25]
VFG0430		Vi antigen	*tviC*	Vi polysaccharide biosynthesis protein, epimerase	*E. anophelis*<br>*E. miricola*	[1]
VFG0428			*tviE*	Vi polysaccharide biosynthesis protein TviE, Glycosyl transferases group 1	*E. anophelis*	[25]
VFG0431			*tviB*	Vi polysaccharide biosynthesis protein, UDP-glucose/GDP-mannose dehydrogenase	*E. meningoseptica*<br>*E. anophelis*<br>*E. miricola*	[1,5,10,25,27]
	Invasion	Adherence; Porin	*DnaK*	Molecular chaperone	*E. anophelis*	[16]
VFG1444		AslA	*aslA*	Putative arylsulfatase	*E. anophelis*	[25]
VFG0070		p60	*iap*	P60 extracellular protein, invasion associated protein Iap	*E. anophelis*	[25]
VFG2526	Motility	Flagella	*cheB*	Chemotaxis-specific methylesterase	*E. anophelis*	[25]

VFG2532			*cheY1*	Chemotaxis two-component response regulator CheY1	*E. anophelis*	[25]
VFG011946			*flgR*	Sigma-54 associated transcriptional activator	*E. anophelis*	[10]
VFG011850			*flhG*	ATP-binding protein	*E. anophelis*<br>*E. miricola*	[1]
VFG043385<br>VFG011850			*ylxH*	ATP-binding protein	*E. anophelis*	[10]
VFG001249			*fleR*	Two-component response regulator	*E. meningoseptica*<br>*E. anophelis*<br>*E. miricola*	[1,10]
VFG2008			*MotB*	Flagellar motor protein	*E. meningoseptica*<br>*E. anophelis*<br>*E. miricola*	[6,25]
VFG1922			*ptmA*	Putative oxidoreductase (flagellin modification)	*E. anophelis*	[25]
VFG1243		Polar flagella	*flgJ*	Flagellar rod assembly protein	*E. anophelis*	[25]
			flmH	Short chain dehydrogenase/reductase family oxidoreductase	*E. anophelis*<br>*E. miricola*<br>*E. ursingii*<br>*E. occulta*	[16,20,26]
VFG1248			*fleQ*	Transcriptional regulator	*E. meningoseptica*<br>*E. anophelis*	[1,5,10,25,27]

					E. miricola	
VFG1237			*FlgD*	Flagellar hook capping protein	*E. meningoseptica* *E. anophelis* *E. miricola*	[6]
VFG0937	Nutritional/Metabolic factor	Aerobactin	*iucD*	L-lysine 6-monooxygenase IucD	*E. anophelis*	[25]
VFG1085			*iutA*	Ferric aerobactin receptor precusor IutA	*E. anophelis*	[25]
VFG0917		Chu	*chuA*	Outer membrane heme/hemoglobin receptor ChuA	*E. anophelis*	[25]
VFG0916			*chuS*	Heme oxygenase ChuS	*E. anophelis*	[25]
VFG0922			*chuU*	Heme permease protein ChuU	*E. anophelis*	[25]
VFG044172			*chuV*	ATP-binding hydrophilic protein	*E. meningoseptica* *E. anophelis* *E. miricola*	[1]
VFG0934		Enterobactin	*entA*	2,3-dihydro-2,3-dihydroxybenzoate dehydrogenase	*E. anophelis*	[25]
VFG000923			*fepA*	Ferrienterobactin outer membrane transporter	*E. anophelis* *E. miricola*	[1,25]
VFG0925			*fepC*	Ferrienterobactin ABC transporter ATPase	*E. anophelis*	[25]

VFG1859	Fe utilization protein A	*feoB*	Fe(2+) transporter permease subunit FeoB	*E. anophelis*	[25]
VFG0344	HitABC	*hitC*	iron(III) ABC transporter, ATP-binding protein	*E. anophelis*	[25]
VFG0358	Yersiniabactin	*fyuA/psn*	pesticin/yersiniabactin receptor protein	*E. anophelis*	[25]
VFG0574	MgtBC	*mgtB*	Mg2+ transport protein	*E. meningoseptica*<br>*E. anophelis*<br>*E. miricola*<br>*E. ursingii*<br>*E. occulta*	[1,5,10,16,20,25–27]
VFG0575	MgtC	*mgtC*	Possible Mg2+ transport P-type ATPase C	*E. meningoseptica*<br>*E. anophelis*<br>*E. miricola*	[1,5,10,25,27]
VFG9570	Mycobacti	*irtA*	Iron-regulated transporter	*E. meningoseptica*<br>*E. anophelis*<br>*E. miricola*	[1,10]
VFG0168	Pyochelin	*pchD*	Pyochelin biosynthesis protein PchD	*E. anophelis*	[25]
VFG1267	Pyochelin	*pchH*	ABC transporter ATP-binding protein	*E. anophelis*	[25]
VFG1266	Pyochelin	*pchI*	ABC transporter ATP-binding protein	*E. anophelis*	[25]

VFG0165		Pyochelin	*pchF*	Pyochelin synthetase	*E. meningoseptica*<br>*E. anophelis*<br>*E. miricola*	[1,10]
VFG0167		Pyochelin	*pchR*	Transcriptional regulator	*E. meningoseptica*<br>*E. anophelis*<br>*E. miricola*	[1,5,25]
VFG0160		Pyoverdine	*pvdE*	Pyoverdine biosynthesis protein	*E. meningoseptica*<br>*E. anophelis*<br>*E. miricola*	[1,5,10,25]
VFG1650		Salmochelin	*iroN*	Salmochelin receptor IroN	*E. anophelis*	[25]
VFG1653		Salmochelin	*iroC*	ATP binding cassette transporter	*E. meningoseptica*<br>*E. anophelis*<br>*E. miricola*	[1,25]
VFG0366		Yersiniabactin	*ybtQ*	Inner membrane ABC-transporter	*E. meningoseptica*<br>*E. anophelis*<br>*E. miricola*	[1,10,25,27]
VFG0364		Yersiniabactin	*ybtA*	putative AraC type regulator	*E. anophelis*	[25]
VFG0365		Yersiniabactin	*ybtP*	Putative inner membrane ABC-transporter	*E. anophelis*	[25]
VFG001864	Post-translational modification	Macrophage infectivity potentiator	*mip*	Macrophage infectivity potentiator	*E. meningoseptica*<br>*E. anophelis*<br>*E. miricola*	[1,5,10,25,27]
VF0449		PrsA2	*prsA2*	Post translocation chaperone	*E. meningoseptica*<br>*E. anophelis*	[1]

VFC0315					*E. miricola*	
VFG2044	Regulation	BvgAS	*bvgA*	Virulence factors transcription regulator	*E. anophelis*	[25]
VFG0478		Ferric uptake regulator	*fur*	Transcriptional repressor of iron-responsive genes (Fur family) (ferric uptake regulator)	*E. anophelis*	[25]
VFG1406		Iron-dependent regulator	*ideR*	Iron-dependent repressor and activator IdeR	*E. anophelis*	[25]
VFG1889		LetA/S	*letA*	Response regulator GacA	*E. anophelis*	[25]
VFG1390		Mycobacterial persistence regulator (MprAB)	*mprA*	Two component response transcriptional regulatory protein MprA	*E. anophelis*	[25]
VFG1887		RelA	*relA*	GTP pyrophosphokinase ((p)ppGpp synthetase I) stringent stress response RelA	*E. anophelis*	[25]
VFG1866		RpoS	*rpoS*	RNA polymerase sigma factor RpoS	*E. anophelis*	[5,25,27]
VFG1412		SigH	*sigH*	RNA polymerase sigma-E factor	*E. anophelis*	[25]
VFG1404	Stress survival	AhpC	*ahpC*	Putative alkylhydroperoxidase C	*E. anophelis*	[25]
VFG000077		clpP	*clpP*	ATP-dependent Clp protease proteolytic subunit	*E. meningoseptica*<br>*E. anophelis*<br>*E. miricola*	[1,5,10,16,25,27]

VFG0080		ClpE	*clpE*	Fimbrial chaperone protein	*E. anophelis*	[5,10,25]
VFG1867		SodB	*sodB*	Superoxide dismutase	*E. meningoseptica*<br>*E. anophelis*<br>*E. miricola*	[1,5,10,25,27]
VFG1861		KatAB	*katA*	Catalase/(hydro)peroxidase	*E. meningoseptica*<br>*E. anophelis*<br>*E. miricola*<br>*E. bruuniana*<br>*E. ursingii*<br>*E. occulta*	[1,5,10,16,20,25–27]
VFG1396		KatG	*katG*	Catalase/peroxidase HPI	*E. meningoseptica*<br>*E. anophelis*<br>*E. miricola*<br>*E. bruuniana*<br>*E. ursingii*<br>*E. occulta*	[10,16,20,26]
VFG0274		Urease	*ureG*	Urease accessory protein	*E. meningoseptica*<br>*E. anophelis*<br>*E. miricola*<br>*E. ursingii*<br>*E. occulta*	[1,20,26]
VFG0269		Urease	*ureA*	Urease alpha subunit	*E. miricola*	[1]
VFG0272		Urease	*ureE*	Urease accessory protein	*E. miricola*	[1]
VFG0869	Others	Dispersin	*aatC*	ATP-binding protein AatC	*E. anophelis*	[5,25,27]

VFG1381	Isocitrate lyase	*icl*	Isocitrate lyase Icl (isocitrase) (isocitratase)	*E. meningoseptica*<br>*E. anophelis*<br>*E. miricola*<br>*E. bruuniana*<br>*E. ursingii*<br>*E. occulta*	[1,5,10,16,20,26]
VFG1731	c3610	*c3610*	Unknown	*E. anophelis*	[25]
	Desferrioxamine	*dfoA*	L-lysine 6-monooxygenase involved in desferrioxamine biosynthesis	*E. anophelis*	[16]
		dfoC	Desferrioxamine siderophore biosynthesis protein dfoC	*E. anophelis*	[16]
		dfoJ	Putative decarboxylase involved in desferrioxamine biosynthesis	*E. anophelis*	[16]
	Heme biosynthesis	*hemL*	Glutamate-1-semialdehyde aminotransferase	*E. meningoseptica*<br>*E. anophelis*<br>*E. miricola*<br>*E. ursingii*<br>*E. occulta*	[16,20,26]
VFG1411	Unknown	*leuD*	3-isopropylmalate dehydratase small subunit	*E. anophelis*	[5,25]
VFG037100	Methionine sulphoxide reductase (MsrAB)	*msrB_2*	Trifunctional thioredoxin/methionine sulfoxide reductase A/B protein	*E. meningoseptica*<br>*E. anophelis*<br>*E. miricola*	[1,10,16]

	N-linked protein glycosylation	*pglC*	General glycosylation pathway protein	*E. anophelis*	[16]
	P. syringae TTSS effectors	*hopJ1*	Type III effector HopJ1	*E. miricola*<br>*E. ursingii*<br>*E. occulta*	[20]
VFG1389	PrrA/B	*prrA*	Transcriptional regulatory protein PrrA	*E. anophelis*	[25]
	Streptococcal enolase	*eno*	Phosphopyruvate hydratase	*E. meningoseptica*<br>*E. anophelis*<br>*E. miricola*<br>*E. bruuniana*<br>*E. ursingii*<br>*E. occulta*	[16,20,26]
VFG1539	Hek	*hek*	Adhesin/virulence factor Hek	*E. anophelis*	[25]
VFG1398		*trpD*	Anthranilate phosphoribosyltransferase	*E. anophelis*	[25]
VFG1068	Unknown	*orf52*	Hypothetical protein	*E. anophelis*	[5]
VFG1706	Unknown	*c3575*	Transposase insF for insertion sequence IS3A/B/C/D/E/fA	*E. anophelis*	[25]
VFG1722	Unknown	*c3601*	Hypothetical protein	*E. anophelis*	[25]
VFG0562	Unknown	*mutS*	methyl-directed mismatch repair, recognize exocyclic adducts of guanosine	*E. anophelis*	[25]
VFG1473	Unknown	*ORF22*	hypothetical protein	*E. anophelis*	[25]

VFG0491	Unknown	*orf242*	putative regulatory proteins, merR family	*E. anophelis*	[25]
VFG1050	Unknown	*orf34*	Unkown	*E. anophelis*	[25]
VFG1054	Unknown	*orf38*	Hypothetical protein	*E. anophelis*	[25]
VFG0483	Unknown	*orf408*	putative regulatory protein, deoR family	*E. anophelis*	[25]
VFG1580	Unknown	*orf46*	Hypothetical protein	*E. anophelis*	[25]
VFG1583	Unknown	*orf49*	hypothetical protein	*E. anophelis*	[25]
VFG1456	Unknown	*ORF5*	hypothetical protein	*E. anophelis*	[25]
VFG1074	Unknown	*orf58*	IS1328 transposase	*E. anophelis*	[25]
VFG1512	Unknown	*ORF61*	putative reverse transcriptase	*E. anophelis*	[25]
VFG0489	Unknown	*orf7*	Hypothetical protein	*E. anophelis*	[25]
VFG0479	Unknown	*pykF*	pyruvate kinase I (formerly F), fructose stimulated	*E. anophelis*	[25]
VFG0585	Unknown	*soxS*	transcriptional activator of superoxide response regulon (AraC/XylS family)	*E. anophelis*	[25]
VFG0564	Unknown	*sugR*	ATP binding protein	*E. anophelis*	[25]
VFG1036	Unknown	*tetA(B)*	tetracycline resistance protein TetA(B)	*E. anophelis*	[25]

VFG1037	Unknown	*tetC*	putative transcriptional regulator TetC	*E. anophelis*	[25]
VFG1038	Unknown	*tetD*	putative transcriptional regulator TetD	*E. anophelis*	[25]
VFG1030	Unknown	*tnpR*	resolvase TnpR	*E. anophelis*	[25]
VFG0487	Unknown	*ttrS*	Tetrathionate reductase complex: sensory transduction histidine kinase	*E. anophelis*	[25]
VFG1100	Unknown	*VC1767*	conserved hypothetical protein	*E. anophelis*	[25]
VFG0660	Unknown	*yeeS*	intergenic-region protein	*E. anophelis*	[25]
VFG0082	Unknown	*aldA*	Aldehyde dehydrogenase	*E. anophelis*	[5,25]
VFG0087	Unknown	*tagD*	tagD protein	*E. anophelis*	[5,25]
VFG0576	Unknown	*ssb*	ssDNA-binding protein controls activity of RecBCD nuclease	*E. anophelis*	[5,25]
VFG0596	Unknown	*copR*	Copper resistance; transcriptional regulatory protein	*E. anophelis*	[5,25]
VFG1028	Unknown	*intI1*	Tn21 integrase IntI1	*E. anophelis*	[5,25]
VFG1105	Unknown	*VC1772*	Hypothetical protein	*E. anophelis*	[25]
VFG1115	Unknown	*nanK*	ROK family protein	*E. anophelis*	[25]

VFG1511	Unknown	*ORF60*	Putative integrase	*E. anophelis*	[5,25]
VFG1584	Unknown	*orf50*	Hypothetical protein	*E. anophelis*	[5,25]
VFG1587	Unknown	*orf53*	Hypothetical protein	*E. anophelis*	[5,25]
VFG1668	Unknown	*orf45*	Putative lysil-tRNA synthetase LysU	*E. anophelis*	[5,25]
VFG1633	Unknwon	*mchF*	Microcin transport protein MchF	*E. anophelis*	[25]
VFG1051	Unknwon	*ORF35*	Putative CS12 fimbrial-like upstream regulatory protein	*E. anophelis*	[25]
VFG0270	Urease	*ureB*	Urease beta subunit UreB, urea amidohydrolase	*E. meningoseptica*<br>*E. anophelis*<br>*E. miricola*<br>*E. ursingii*<br>*E. occulta*	**[1,20,26]**

*Accession number is provided instead.

1. Breurec, S.; Criscuolo, A.; Diancourt, L.; Rendueles, O.; Vandenbogaert, M.; Passet, V.; Caro, V.; Rocha, E.P.; Touchon, M.; Brisse, S. Genomic epidemiology and global diversity of the emerging bacterial pathogen Elizabethkingia anophelis. *Scientific reports* **2016**, *6*, 1-12, doi:10.1038/srep30379.
2. Chen, S.; Soehnlen, M.; Downes, F.; Walker, E. Insights from the draft genome into the pathogenicity of a clinical isolate of *Elizabethkingia meningoseptica* Em3. *Standards in genomic sciences* **2017**, *12*, 56.
3. Amladi, A.; Jacob, J.J.; Anandan, S.; Veeraraghavan, B. Draft genome sequence of carbapenem-resistant Elizabethkingia anophelis strain BP8467 clinical isolate from India. *Journal of global antimicrobial resistance* **2020**, *21*, 200-202, doi:10.1016/j.jgar.2020.04.003.
4. Teo, J.; Tan, S.Y.-Y.; Liu, Y.; Tay, M.; Ding, Y.; Li, Y.; Kjelleberg, S.; Givskov, M.; TP Lin, R.; Yang, L. Comparative Genomic Analysis of Malaria Mosquito Vector-Associated Novel Pathogen Elizabethkingia anophelis. *Genome biology and evolution* **2014**, *6*, 1158-1165, doi:10.1093/gbe/evu094.

5. Li, Y.; Liu, Y.; Chew, S.C.; Tay, M.; Salido, M.M.S.; Teo, J.; Lauro, F.M.; Givskov, M.; Yang, L. Complete genome sequence and transcriptomic analysis of the novel pathogen Elizabethkingia anophelis in response to oxidative stress. *Genome biology and evolution* **2015**, *7*, 1676-1685, doi:10.1093/gbe/evv101.
6. Chen, S.; Soehnlen, M.; Blom, J.; Terrapon, N.; Henrissat, B.; Walker, E.D. Comparative genomic analyses reveal diverse virulence factors and antimicrobial resistance mechanisms in clinical Elizabethkingia meningoseptica strains. *PloS one* **2019**, *14*, e0222648, doi:10.1371/journal.pone.0222648.
7. Yum, J.H.; Lee, E.Y.; Hur, S.-H.; Jeong, S.H.; Lee, H.; Yong, D.; Chong, Y.; Lee, E.-W.; Nordmann, P.; Lee, K. Genetic diversity of chromosomal metallo-β-lactamase genes in clinical isolates of Elizabethkingia meningoseptica from Korea. *The Journal of Microbiology* **2010**, *48*, 358-364, doi:10.1007/s12275-010-9308-5.
8. González, L.J.; Vila, A.J. Carbapenem resistance in Elizabethkingia meningoseptica is mediated by metallo-β-lactamase BlaB. *Antimicrobial agents and chemotherapy* **2012**, *56*, 1686-1692.
9. Lau, S.K.; Wu, A.K.; Teng, J.L.; Tse, H.; Curreem, S.O.; Tsui, S.K.; Huang, Y.; Chen, J.H.; Lee, R.A.; Yuen, K.-Y. Evidence for Elizabethkingia anophelis transmission from mother to infant, Hong Kong. *Emerging infectious diseases* **2015**, *21*, 232, doi:10.3201/eid2102.140623.
10. Perrin, A.; Larsonneur, E.; Nicholson, A.C.; Edwards, D.J.; Gundlach, K.M.; Whitney, A.M.; Gulvik, C.A.; Bell, M.E.; Rendueles, O.; Cury, J. Evolutionary dynamics and genomic features of the Elizabethkingia anophelis 2015 to 2016 Wisconsin outbreak strain. *Nature communications* **2017**, *8*, 1-12, doi:10.1038/ncomms15483.
11. Bellais, S.; Aubert, D.; Naas, T.; Nordmann, P. Molecular and biochemical heterogeneity of class B carbapenem-hydrolyzing β-lactamases in Chryseobacterium meningosepticum. *Antimicrobial agents and chemotherapy* **2000**, *44*, 1878-1886, doi:10.1128/AAC.44.7.1878-1886.2000.
12. Matyi, S.A.; Hoyt, P.R.; Hosoyama, A.; Yamazoe, A.; Fujita, N.; Gustafson, J.E. Draft genome sequences of Elizabethkingia meningoseptica. *Genome announcements* **2013**, *1*, e00444-00413, doi:10.1128/genomeA.00444-13.
13. Sun, G.; Wang, L.; Bao, C.; Li, T.; Ma, L.; Chen, L. Complete genome sequence of Elizabethkingia meningoseptica, isolated from a T-cell non-Hodgkin's lymphoma patient. *Genome announcements* **2015**, *3*, e00673-00615, doi:10.1128/genomeA.00673-15.
14. Hu, R.; Zhang, Q.; Gu, Z. Whole-genome analysis of the potentially zoonotic Elizabethkingia miricola FL160902 with two new chromosomal MBL gene variants. *Journal of Antimicrobial Chemotherapy* **2020**, *75*, 526-530, doi:10.1093/jac/dkz480.
15. Steinberg, J.P.; Burd, E.M. Other gram-negative and gram-variable bacilli. *Principles and practice of infectious diseases* **2010**, *2*, 2751-2768.
16. Wang, M.; Gao, H.; Lin, N.; Zhang, Y.; Huang, N.; Walker, E.D.; Ming, D.; Chen, S.; Hu, S. The antibiotic resistance and pathogenicity of a multidrug-resistant Elizabethkingia anophelis isolate. *MicrobiologyOpen* **2019**, *8*, e804, doi:10.1002/mbo3.804.
17. Rossolini, G.M.; Franceschini, N.; Lauretti, L.; Caravelli, B.; Riccio, M.L.; Galleni, M.; Frère, J.-M.; Amicosante, G. Cloning of a Chryseobacterium (Flavobacterium) meningosepticum chromosomal gene (blaA CME) encoding an extended-spectrum class A β-

lactamase related to the Bacteroides cephalosporinases and the VEB-1 and PER β-lactamases. *Antimicrobial Agents and Chemotherapy* **1999**, *43*, 2193-2199, doi:10.1128/AAC.43.9.2193.
18. Ghafoori, S.M.; Robles, A.M.; Arada, A.M.; Shirmast, P.; Dranow, D.M.; Mayclin, S.J.; Lorimer, D.D.; Myler, P.J.; Edwards, T.E.; Kuhn, M.L. Structural characterization of a Type B chloramphenicol acetyltransferase from the emerging pathogen Elizabethkingia anophelis NUHP1. *Scientific reports* **2021**, *11*, 1-10, doi:10.1038/s41598-021-88672-z.
19. Chen, C.; Chen, Y.; Wang, F. Risk factors of healthcare-associated *Elizabethkingia meningoseptica* infections in Taiwan medical center. *International Journal of Antimicrobial Agents* **2017**, *50*, S141-S141.
20. Liang, C.-Y.; Yang, C.-H.; Lai, C.-H.; Huang, Y.-H.; Lin, J.-N. Comparative Genomics of 86 Whole-Genome Sequences in the Six Species of the Elizabethkingia Genus Reveals Intraspecific and Interspecific Divergence. *Scientific reports* **2019**, *9*, 1-11, doi:10.1038/s41598-019-55795-3.
21. Naidenov, B.; Lim, A.; Willyerd, K.; Torres, N.J.; Johnson, W.L.; Hwang, H.J.; Hoyt, P.; Gustafson, J.E.; Chen, C. Pan-genomic and polymorphic driven prediction of antibiotic resistance in Elizabethkingia. *Frontiers in microbiology* **2019**, *10*, 1446, doi:10.3389/fmicb.2019.01446.
22. Lin, J.-N.; Lai, C.-H.; Yang, C.-H.; Huang, Y.-H.; Lin, H.-H. Clinical manifestations, molecular characteristics, antimicrobial susceptibility patterns and contributions of target gene mutation to fluoroquinolone resistance in Elizabethkingia anophelis. *Journal of Antimicrobial Chemotherapy* **2018**, *73*, 2497-2502, doi:10.1093/jac/dky197.
23. Johnson, W.L.; Ramachandran, A.; Torres, N.J.; Nicholson, A.C.; Whitney, A.M.; Bell, M.; Villarma, A.; Humrighouse, B.W.; Sheth, M.; Dowd, S.E. The draft genomes of Elizabethkingia anophelis of equine origin are genetically similar to three isolates from human clinical specimens. *PloS one* **2018**, *13*, e0200731, doi:10.1371/journal.pone.0200731.
24. Jiang, X.; Wang, D.; Wang, Y.; Yan, H.; Shi, L.; Zhou, L. Occurrence of antimicrobial resistance genes sul and dfrA 12 in hospital environmental isolates of Elizabethkingia meningoseptica. *World Journal of Microbiology and Biotechnology* **2012**, *28*, 3097-3102.
25. Teo, J.; Tan, S.Y.-Y.; Liu, Y.; Tay, M.; Ding, Y.; Li, Y.; Kjelleberg, S.; Givskov, M.; Lin, R.T.; Yang, L. Comparative genomic analysis of malaria mosquito vector-associated novel pathogen Elizabethkingia anophelis. *Genome biology and evolution* **2014**, *6*, 1158-1165.
26. Yang, C.; Liu, Z.; Yu, S.; Ye, K.; Li, X.; Shen, D. Comparison of Whole-Genome Sequences for Three Species of the Elizabethkingia Genus. **2020**.
27. Lin, J.-N.; Lai, C.-H.; Yang, C.-H.; Huang, Y.-H.; Lin, H.-H. Genomic features, phylogenetic relationships, and comparative genomics of Elizabethkingia anophelis strain EM361-97 isolated in Taiwan. *Scientific reports* **2017**, *7*, 1-8, doi:10.1038/s41598-017-14841-8.
